# Synthesis and Herbicidal Activity of Novel *N*-(7-Oxo-4,7-dihydro-[1,2,4]triazolo[1,5-a]pyrimidin-2-yl)arylsulfonamides

**DOI:** 10.3390/molecules31061008

**Published:** 2026-03-17

**Authors:** Xun Li, Yiyi Tian, Xianjun Tang, Jiaqi Li, Huizhe Lu, Xiuhai Gan, Yumei Xiao, Zhaohai Qin

**Affiliations:** 1 College of Science, China Agricultural University, Beijing 100193, China; 2 State Key Laboratory of Green Pesticide, Key Laboratory of Green Pesticide and Agricultural Bioengineering, Ministry of Education, Guizhou University, Guiyang 550025, China

**Keywords:** herbicidal activity, triazolo[1,5-a]pyrimidines, sulfonamides, AHAS inhibitors

## Abstract

Triazolopyrimidine sulfonamide herbicides, a prominent class of acetohydroxyacid synthase (AHAS) inhibitors, are exceptionally effective in controlling weeds in agricultural settings. To overcome metabolic resistance caused by the 5-demethylation of pyroxsulam, we sought to replace its 5-methoxy group on the triazolopyrimidine ring with alkyl substituents. This led to the synthesis of a series of *N*-(7-oxo-4,7-dihydro-[1,2,4]triazolo[1,5-a]pyrimidin-2-yl)arylsulfon-amides, which displayed significant structural diversification potential, culminating in the identification of the herbicidal hit compound **I-20**. However, the suboptimal lipophilicity compromised its herbicidal efficacy. To rectify this limitation, we modified the 7-carbonyl group to a *tert*-butoxy group, resulting in the highly active compound **I-29**. This compound demonstrated herbicidal activity comparable to or exceeding that of penoxsulam against various tested weeds, establishing it as a promising new lead compound and a candidate herbicide for further investigation. Subsequent studies revealed that **I-29** exhibited a receptor binding mode and herbicidal activity profiles that closely aligned with those of penoxsulam. Moreover, its spatial structure was found to be even more conducive to inhibiting flavin adenine dinucleotide (FAD)-mediated AHAS activity. This research not only sheds light on addressing the challenge of 5-demethylation metabolic resistance in triazolopyrimidine sulfonamide herbicides but also offers new avenues for the development of AHAS-inhibiting triazolopyrimidine sulfonamide herbicides.

## 1. Introduction

Acetohydroxyacid synthase (AHAS) inhibitors constitute a class of herbicides that specifically target plant AHAS, thereby impeding the synthesis of branched-chain amino acids [1,2,3,4,5,6,7,8]. Since animals do not possess the biosynthetic pathway for these amino acids and obtain them solely from plant-derived sources, such herbicides exhibit minimal to non-toxic effects on humans and animals [5,8]. Currently, the successful development of AHAS-inhibiting herbicides encompasses various structural types, including sulfonylureas, imidazolinones, triazolopyrimidines, triazolopyrimidine sulfonamides, pyrimidinyloxyphenoxypropionates, triazolinones, pyrimidinyl benzoates, and sulfonamides [9]. As one of the most widely utilized and safest categories of herbicides in contemporary agricultural practice, AHAS inhibitors have consistently maintained a significant presence in the herbicide market due to their high target specificity and favorable environmental compatibility [10].

**Scheme 1:**
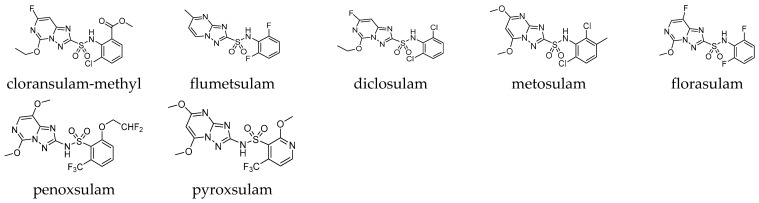
Commercialized triazolopyrimidine herbicides.

Dow AgroSciences is a pioneer in the development of triazolopyrimidine sulfonamide herbicides, with flumetsulam being the first successfully commercialized product in this category [3,11,12,13,14]. By inhibiting the activity of acetohydroxyacid synthase (AHAS), flumetsulam disrupts the synthesis of branched-chain amino acids—specifically leucine, valine, and isoleucine—thereby hindering protein synthesis in plants. This action leads to growth arrest and ultimately results in weed mortality. Common injury symptoms in affected weeds include vein chlorosis, leaf fading, blanching or purpling, shortened internodes due to meristem inhibition, and apical bud death. Flumetsulam is effective for controlling a wide range of both annual and perennial broadleaf weeds in corn, soybean, wheat, and other crop fields, while also exhibiting some inhibitory effects on young grassy weeds [15,16]. Currently, commercial triazolopyrimidine herbicides can be categorized into two groups, triazolopyrimidine sulfonamides and sulfonamidotriazolopyrimidines, which are functional group isomers of one another (Scheme 1). The core triazolopyrimidine structure exists in two variants: [1,5-a] and [1,5-c]. These herbicides play a significant role in weed management. For example, metosulam is utilized for the post-emergence control of numerous important broadleaf weeds in corn, wheat, barley, and rye fields [3]. Cloransulam-methyl is specifically applied for broadleaf weed control in soybean fields [17]. Diclosulam is employed for pre-emergence and soil treatment in soybean and peanut fields, targeting broadleaf weeds [18]. Florasulam effectively controls most broadleaf weeds in wheat fields, including challenging species such as *Galium aparine* and *Lithospermum arvense*, and demonstrates excellent inhibitory activity against *Euphorbia helioscopia*, a particularly troublesome weed in such environments [19]. Penoxsulam is effective in managing *Echinochloa crus-galli*, *Leptochloa chinensis*, and various annual cyperaceous weeds in paddy fields, as well as a range of broadleaf weeds [4,20]. Pyroxsulam exhibits high efficacy against common grassy and broadleaf weeds and shows activity against resistant *Alopecurus japonicus* and other problematic species [8]. Given the widespread use of prominent herbicides such as flumetsulam and penoxsulam, triazolopyrimidine sulfonamide herbicides have garnered increasing attention and remain a vital focus for research and development in herbicide innovation [21].

**Figure 1:**
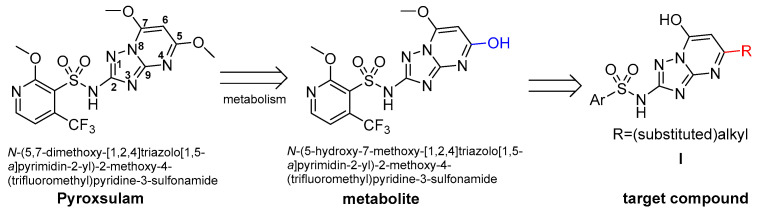
Design strategy of the title compounds.

Triazolopyrimidine sulfonamide herbicides typically comprise three components: a triazolopyrimidine heterocyclic core, a sulfonamide bridge, and a substituted aromatic ring [22]. Currently, fewer than ten triazolopyrimidine sulfonamide herbicides are commercially available worldwide. The limitations imposed by structural modification bottlenecks in traditional synthetic pathways hinder the development of new candidates to address the growing weed resistance and meet the demand for multi-crop adaptability, especially for simultaneous application in rice, wheat, and soybean fields [8,23,24]. From a chemical perspective, existing commercial triazolopyrimidine sulfonamide herbicides share highly similar structural scaffolds, leading to rapid evolution of cross-resistance in weed populations. The lack of novel skeletons and functional group diversification has become a major bottleneck for the innovation of this herbicide category. Consequently, there is an urgent need to address the challenge of insufficient chemical diversity through innovative synthetic processes. According to Satchivi, pyroxsulam is metabolically demethylated in plants primarily to form inactive 5-hydroxypyroxsulam, a detoxification pathway considered the main route for pyroxsulam’s degradation [25,26,27]. To counteract this metabolic resistance and facilitate the development of herbicides within this class that possess greater structural diversification potential, we aimed to replace the 5-methoxy group with alkyl groups and synthesized the target compounds **I** (Figure 1). This approach is intended to provide a structural foundation for developing novel, high-efficiency, broad-spectrum, and low-resistance herbicides [25].

## 2. Results and Discussion

### 2.1. Chemistry

**Figure 2:**
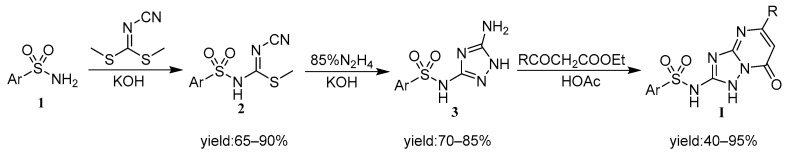
Synthetic pathway of compound **I**.

**Figure 3:**
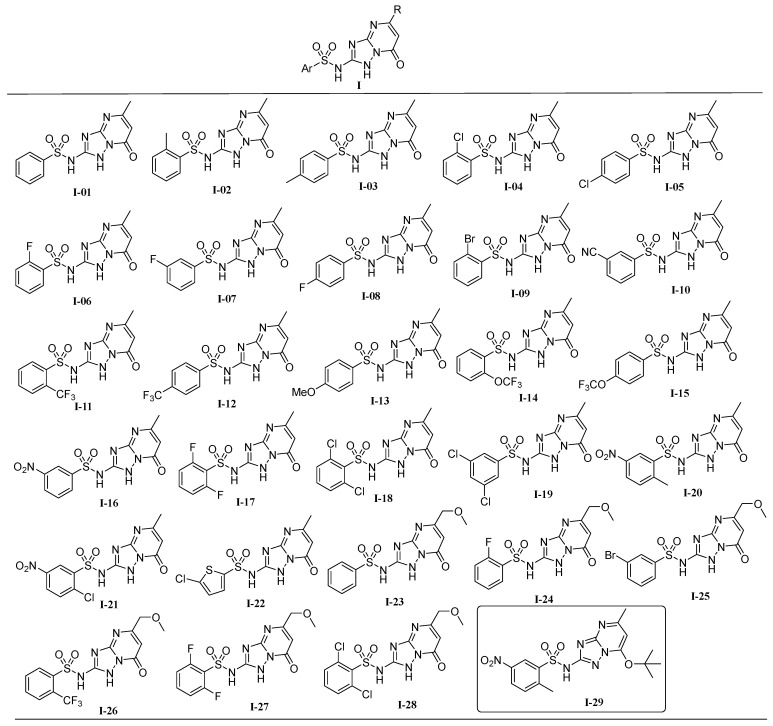
Structures of **I** compounds and **I-29**.

As a representative example of [1,5-a]triazolopyrimidine herbicides, the classic synthetic route for pyroxsulam involves the initial construction of a 2-aminotriazolopyrimidine skeleton, followed by sulfonylation with sulfonyl chloride [9]. Although this method has been industrialized, it presents significant limitations: the regioselectivity of sulfonylation is challenging to control precisely, and achieving molecular structural diversification is difficult [28]. To address these limitations, this study proposes a novel synthetic strategy that utilizes sulfonamide as the starting material. This approach involves the initial construction of the triazole ring, followed by the formation of the triazolopyrimidine skeleton (Figure 2). This method successfully integrates precise regioselectivity with enhanced derivatization flexibility, offering a new perspective for the efficient synthesis and structural optimization of novel triazolopyrimidine sulfonamide herbicides. The compounds synthesized through this innovative route are presented in Figure 3.

### 2.2. Single-Crystal Diffraction Analysis

**Figure 4:**
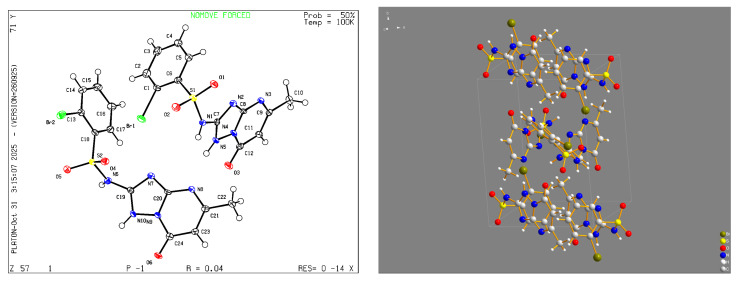
Crystal structure and packing diagram of compound **I-09** (CCDC: 2499604).

Triazolopyrimidinone compounds may theoretically exhibit keto-enol tautomerism in solution. To clarify their stable existence form in the solid state, single crystal cultivation of compound **I-09** was carried out in a mixed solvent system (H_2_O-MeOH-DCM-Hexane, pH ≈ 5~7) and an aprotic solvent system (DMSO-EA), respectively. The single crystal diffraction results obtained in the two solvent systems were completely consistent, corresponding to the same configuration (Figure 4, left). As can be seen from the unit cell packing diagram (Figure 4, right), the compound molecules are packed in a three-dimensional network or layered interweaving pattern in the unit cell, rather than a simple one-dimensional chain or two-dimensional layered structure. The molecule adopts a stable right-angle conformation in the solid state, with the core corner at the connection site between the heterocyclic core and the sulfonamide side chain, and the spatial angle formed by the rigid fragments on both sides is close to 90°.

### 2.3. Herbicidal Activity

Table 1 lists the preliminary herbicidal activity results of **I** compounds and penoxsulam against three representative weeds: *E. crus-galli* (Gramineae), *S. cannabina* (broadleaf) and *A. theophrasti* (broadleaf). Overall, at a dosage of 70 g a.i./ha, the activity of the target compounds against the three types of weeds followed the order of *A. theophrasti* > *S. cannabina* > *E. crusgalli*. Most compounds exhibited high activity against *A. theophrasti* and *S. cannabina*, while only a few compounds showed simultaneous high activity against *E. crusgalli*, indicating that broadleaf weeds are more sensitive to this class of compounds, and there are certain differences in the sensitivity of different broadleaf weeds to these compounds. By comparing the compound pairs **I-01**/**I-23**, **I-06**/**I-24**, **I-11**/**I-26**, **I-17**/**I-27** and **I-18**/**I-28**, no significant difference in activity was observed between each pair, suggesting that R = CH_3_ or CH_2_OCH_3_ has little effect on activity, and the volume of this group is not a key factor affecting the activity of the target compounds [22]. For the Ar moiety, substitution on the phenyl ring (whether electron-donating or electron-withdrawing groups) is generally beneficial to the activity. Among all monosubstituted compounds, although substituents of different types and positions showed certain activity differences, such differences were not particularly prominent. Only in monohalogenated aryls, ortho-halogenated compounds were generally superior to those with substitutions at other positions, showing obvious substituent position effects [22,29]. Interestingly, 2,6-dihalogenated compounds (**I-17**, **I-18**, **I-27** and **I-28**), a characteristic structure of commercial triazolopyrimidine herbicides, did not exhibit particularly prominent activity different from other compounds. However, the significantly higher activity of **I-18** than **I-19** still indicates the important influence of substituent position.

A significant and unexpected finding of this study is that compound **I-20** exhibited markedly superior activity compared to other tested compounds. It demonstrated excellent efficacy not only against broadleaf weeds but also a substantial inhibitory effect on the gramineous weed *E. crus-galli*. Although its overall activity was lower than that of the control penoxsulam, it captured our interest, leading to the selection of **I-20** as a hit compound for further investigation.

**Figure 5:**
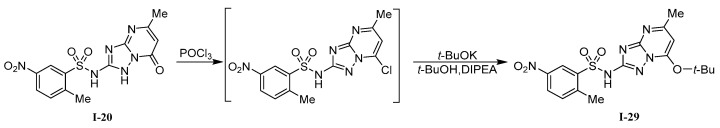
Synthetic pathway of compound **I-29**.

Our original design was to synthesize 7-hydroxy-substituted triazolopyrimidines with the identical triazolopyrimidine heterocyclic structure to pyroxsulam. The formation of 7-hydroxypyroxsulam is also one of the minor metabolic pathways of pyroxsulam, and the study of these compounds is also helpful to clarify whether the 7-demethylation metabolic pathway is a detoxification pathway of pyroxsulam. However, the single crystal diffraction results of **I-09** showed that this class of compounds is actually the keto tautomer of its enol form, resulting in an obvious structural difference between them and pyroxsulam in the triazolopyrimidine heterocyclic structure. To reveal the effect of this structural difference on activity, we calculated and compared the logP values of **I-20** and existing triazolopyrimidine herbicides using ACD Labs (free trial version, Advanced Chemistry Development Inc., Toronto, ON, Canada). The results showed that the logP value of the keto form of **I-20** was 0.59 ± 0.78, which is significantly lower than the suitable range of 1.0~4.0 for herbicide development, while the logP values of penoxsulam, cloransulam-methyl and diclosulam were 2.87 ± 1.30, 3.52 ± 0.79 and 2.88 ± 0.73, respectively (Table S3). This difference in the triazolopyrimidine ring and logP value is likely a key factor affecting its activity. To address this, we converted the 7-carbonyl group of **I-20** to a *tert*-butoxy group via chlorination followed by substitution to synthesize compound **I-29** (Figure 5) and re-evaluated its activity in comparison with **I-20**. The results are shown in Table 2 and Figures S1 and S2.

The results showed that compared with **I-20**, to our satisfaction, the herbicidal activity of **I-29** was comprehensively improved. At a dosage of 30 g a.i./ha, the growth inhibition rate of **I-29** against all tested weeds reached more than 90% (activity grade +++++), characterized by complete chlorosis and necrosis of the whole weed plant, chlorosis and lignification of roots, lodging and complete loss of growth activity. At this dosage, **I-20** also exhibited complete inhibition (+++++) on broadleaf weeds (*S. cannabina*, *A. theophrasti*, and *E. prostrata*), but only slight wilting at the seedling growing points (+) on the gramineous weed *D. sanguinalis*, with the main plant parts still maintaining normal growth without obvious chlorosis or lodging. At a dosage of 15 g a.i./ha, the difference in herbicidal activity between the compounds became more prominent: **I-29** still maintained high activity (+++++ and ++++) against all tested weeds, with *E. crusgalli* and *E. indica* showing complete necrosis and lignification, chlorosis and lodging, *E. prostrata* showing whole plant blackening without growth signs, and only a few *D. sanguinalis* plants standing upright without lodging, with the overall inhibitory effect not significantly different from that at the high dosage. In contrast, **I-20** still maintained high inhibitory activity (+++++) on broadleaf weeds at this dosage, but its inhibitory activity on gramineous weeds decreased significantly: the main parts of *E. crusgalli* plants were green with only chlorosis at the growing points (+++), about half of *E. indica* plants were chlorotic and lodged (++), and *D. sanguinalis* showed no obvious inhibitory effect (-), almost the same as the blank control. The positive control penoxsulam showed a slight decrease in inhibitory activity on *E. crusgalli* and *E. indica* at this dosage (+++++) and only wilting at the growing points (+) on *D. sanguinalis*, with an inhibitory effect significantly weaker than that of **I-29** (Figure S1). At the low dosage of 7.5 g a.i./ha, the herbicidal activity of all compounds decreased to varying degrees, but **I-29** still maintained a significant activity advantage, with the inhibition rate of all tested weeds remaining above 70% (+++++): the roots of *E. crusgalli* still retained a small amount of green but the main plant parts were chlorotic (+++++), only a small number of *E. indica* plants were not completely chlorotic with partial green tissues remaining (+++), *D. sanguinalis* showed lodging without lignification and necrosis (+++) and still had certain growth potential, and a large area of *E. prostrata* was black and necrotic with only a small number of plants surviving (+++++). In contrast, the activity of **I-20** attenuated sharply at this dosage: only a small number of plants showed chlorosis at the growing points (++) on *E. crusgalli* and *S. cannabina*, about half of *E. indica* plants still grew normally (+++), *D. sanguinalis* showed no inhibitory effect (-), and only a small number of *E. prostrata* plants turned black while most developed normally (++). The inhibitory activity of penoxsulam on gramineous weeds further decreased at this dosage, showing only slight wilting at the growing points (+) on *D. sanguinalis* and inhibiting the growth of only about half of *E. prostrata* plants (+++), which was significantly weaker than **I-29** (Figure S1).

In terms of target weed types, the broadleaf weeds *S. cannabina* and *A. theophrasti* showed inherent sensitivity to the tested triazolopyrimidine sulfonamide compounds. **I-20** could achieve complete inhibition at medium and high dosages, while **I-29** still maintained high-efficiency inhibition at low dosages, which was comparable to the activity of penoxsulam. There were large differences in the sensitivity of gramineous weeds to this class of compounds: *D. sanguinalis* showed obvious tolerance to **I-20**, while **I-29** broke through this limitation and achieved high-efficiency inhibition of intractable gramineous weeds such as *D. sanguinalis* and *E. indica* at medium and low dosages, a characteristic even significantly superior to the positive control penoxsulam. In summary, **I-29** not only inherited the high-efficiency inhibitory activity of the hit compound **I-20** on broadleaf weeds but also significantly improved the activity on gramineous weeds, exhibiting broad-spectrum and high-efficiency herbicidal characteristics with activity close to or even superior to penoxsulam [14]. However, since only principle verification was conducted here, although **I-29** has the characteristics of a candidate herbicide, the possibility of further optimizing and screening **I-29** as a new lead compound to develop a new generation of AHAS-inhibiting triazolopyrimidine herbicides with better performance still exists, which will be the goal of our next research.

### 2.4. AHAS Inhibitory Activity

**Figure 6:**
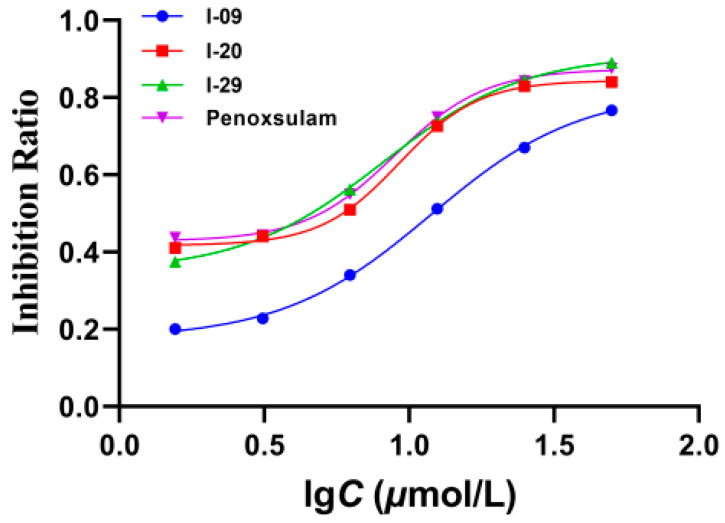
Logarithm of compound concentration [log C (μM)] vs. AHAS enzyme inhibition (0–1 scale). Data points represent mean values from triplicate experiments, and curves are fitted with a four-parameter logistic model by GraphPad Prism (free trial).

To reveal the molecular mechanism of the herbicidal activity differences of this series of compounds and verify their binding ability to the target enzyme, we carried out in vivo AHAS inhibition activity assays and evaluated the low-activity compound **I-09**, hit compound **I-20**, lead compound **I-29** and the control agent penoxsulam in parallel. The results are listed in Table 3. The fitting coefficients (R^2^) of the dose–effect relationship curves of all tested compounds were higher than 0.99, indicating excellent fitting quality and high reliability of the experimental data. The inhibitory activity of compound **I-20** was close to that of penoxsulam, which may be due to the use of the germinated root parts of *E. crusgalli* with a thin cuticle. This also indirectly confirms that the poor herbicidal activity of **I-20** is likely caused by the difficulty of the inhibitor molecules to penetrate the plant cuticle due to insufficient lipophilicity. The derivative **I-29** obtained by structural modification exhibited the strongest enzyme inhibitory activity with an IC_50_ value of 8.09 ± 0.09 μM, which was not only significantly superior to the low-activity compound **I-09** (11.88 ± 0.10 μM) and the hit compound **I-20** (9.21 ± 0.08 μM) but also slightly better than the control agent penoxsulam (8.44 ± 0.08 μM). **I-29** showed the most prominent target binding ability to the AHAS enzyme. As can be seen from the dose–effect relationship curve (Figure 6), the inhibition rate of **I-29** increased the fastest, and a high level of enzyme inhibition could be achieved at a relatively low concentration, further confirming its better target affinity. Meanwhile, the trend of the dose–effect curves of all tested compounds was highly similar to that of penoxsulam (Figure 6), proving that this series of compounds has the same mechanism of action as penoxsulam, with the AHAS enzyme as the main target. This result is highly consistent with the previous herbicidal activity data: the improvement in the enzyme inhibitory activity of **I-29** is directly related to its broad-spectrum and high-efficiency herbicidal activity, suggesting that structural modification (regulating logP and enhancing lipophilicity) not only optimized the physicochemical properties of the molecule but also further improved its binding efficiency to the target enzyme [29].

### 2.5. Molecular Docking

**Figure 7:**
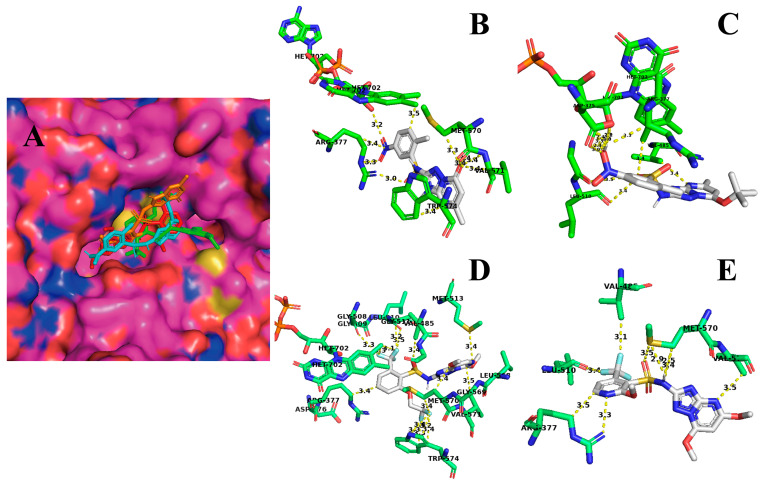
(**A**) Superimposition of **I-20** (orange), **I-29** (blue), penoxsulam (red) and pyroxsulam (green) in the *At*AHAS active pocket [202:A,352:A,377:A,574:A]; (**B**) bond lengths of the interactions between **I-20** and amino acid residues in the *At*AHAS active pocket; (**C**) bond lengths of the interactions between **I-29** and amino acid residues in the *At*AHAS active pocket; (**D**) bond lengths of the interactions between penoxsulam and amino acid residues in the *At*AHAS active pocket; (**E**) bond lengths of the interactions between pyroxsulam and amino acid residues in the *At*AHAS active pocket.

To comprehensively compare the differences in the mode of action of the target compounds **I-20**, **I-29**, penoxsulam and pyroxsulam, we performed molecular docking analysis on the hit and lead compounds in this study with reference to the single crystal structure of the *At*AHAS–penoxsulam complex (PDB: 5WJ1) [16] (Figure 7). Figure 7A clearly shows the binding characteristics of the target compounds **I-20** and **I-29** and the control agents in the *At*AHAS active pocket [202:A,352:A,377:A,574:A], with significant consistency in the binding site conformation of the two compounds. The result confirms that they follow the same *At*AHAS target binding mode and all exert an inhibitory effect by occupying the core active pocket, which is consistent with their AHAS inhibitory activity. From the quantitative molecular docking data (Table S4), the average binding energy of **I-29** reached −8.9 kcal/mol, which was superior to that of **I-20** (−8.7 kcal/mol), penoxsulam (−8.83 kcal/mol) and pyroxsulam (−8.81 kcal/mol). Its optimal conformation (Pose 1) had a binding energy of −9.6 kcal/mol, corresponding to a *K*_i_ of 90.17 nM, which was the strongest in all tested compounds. This data directly confirms that the excellent activity of **I-29** does not stem from the innovation of the binding mode but from the performance upgrade of **I-20** and the control agents through precise structural modification under a conservative binding framework. The key advantages of these compounds stem from focus on the synergistic effect of the *tert*-butoxy group and the meta-nitro group on the regulation of flavin adenine dinucleotide (FAD), a key cofactor of *At*AHAS. As a key structural modification of **I-29** different from **I-20**, the *tert*-butoxy group significantly enhanced the binding stability and spatial orientation of the compound with the active pocket. Due to the lack of a large steric hindrance hydrophobic group, **I-20** can only form a weak electrostatic interaction with the core residue Arg377, with a loose binding conformation (contact area 138 Å^2^) and a conformation fluctuation range of ±0.15 Å, failing to form a stable spatial anchor. Its optimal conformation had a binding energy of −9.2 kcal/mol, corresponding to a *K*_i_ of 182.35 nM and a ligand efficiency of −0.368 kcal/mol/atom, and the binding energy decayed rapidly with conformational changes: the binding energy of Pose 10 was only −8.1 kcal/mol, and the Ki increased to 1182.45 nM, showing poor conformational stability. In contrast, the *tert*-butoxy group of **I-29** formed a strong hydrophobic interaction with Arg377 (interaction distance 3.0–3.2 Å) and a CH-π interaction with Met570 (interaction distance 3.2 Å), constituting a dual hydrophobic anchoring system, which increased the contact area of the molecule in the active pocket to 210 Å^2^ and reduced the conformation fluctuation to only ±0.05 Å, significantly enhancing the binding stability. In terms of quantitative data, the binding energy of **I-29** in all conformations was maintained in the range of −8.4~−9.6 kcal/mol, with a *K*_i_ distribution of 90.17~718.34 nM, and the performance fluctuation between conformations was much smaller than that of **I-20**. More importantly, the spatial steric effect of the *tert*-butoxy group drove the molecular skeleton of **I-29** to shift toward the FAD region, providing a structural basis for the short-range interaction between functional groups and FAD. Notably, the meta-nitro group of **I-29**, as a core functional group, regulated the molecular electron cloud distribution through the electronic inductive effect: on the one hand, it formed a specific hydrogen bond network with Arg377 (bond length 2.98 Å) and His392 (bond length 2.85 Å, 3.02 Å) around FAD; on the other hand, it significantly weakened the electronic repulsion between the molecule and the isoalloxazine ring of FAD. Cooperating with the spatial steric effect of the *tert*-butoxy group, the average distance between **I-29** and FAD was shortened to 3.03 Å (Figure 7B), and the nitro group formed a weak electrostatic interaction with the N5 atom of the FAD isoalloxazine ring (bond length 3.1 Å), further enhancing the regulation efficiency of the FAD oxidation state, which is highly consistent with the conclusion that “FAD oxidation is the key pathway for *At*AHAS inhibition” [30]. In terms of ligand efficiency (LE, LE = binding energy/number of non-hydrogen atoms of the ligand; the more negative the value, the higher the target binding efficiency per atom) [31], the *LE* of **I-29** was in the range of −0.290~−0.331 kcal/mol/atom, which was highly consistent with the high-efficiency range of penoxsulam (−0.277~−0.313) and pyroxsulam (−0.304~−0.332), inheriting the core advantage of per-atom binding of this class of herbicides. Although the optimal conformation *LE* of **I-20** (−0.368) was smaller, this was due to its simpler molecular structure and fewer non-hydrogen atoms. In contrast, while introducing the *tert*-butoxy group to achieve the dual improvement of herbicidal activity and target affinity, **I-29** still maintained a high-efficiency *LE* level comparable to that of the control agents, and the fluctuation between conformations was only 0.041 kcal/mol/atom, smaller than that of **I-20** (0.044 kcal/mol/atom), reflecting the precise balance between functional group modification and binding efficiency.

### 2.6. DFT Calculations

**Figure 8:**
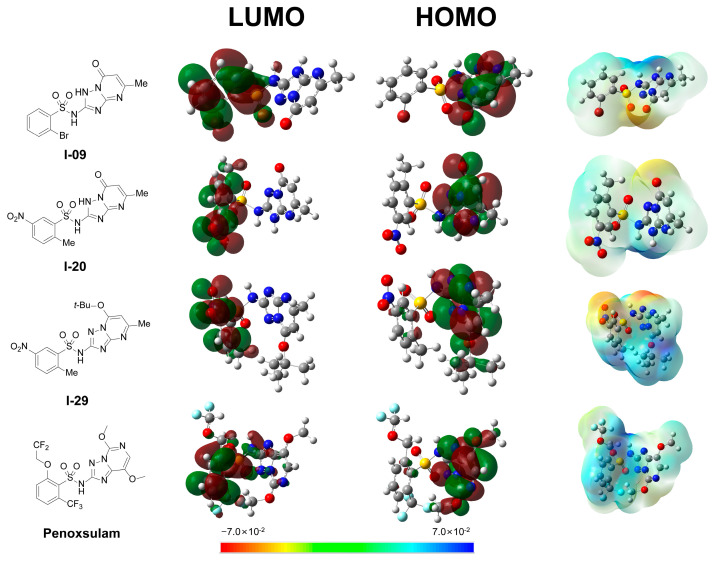
Comparison of van der Waals surface electrostatic potential distributions of **I-09**, **I-20**, **I-29** and penoxsulam.

To systematically reveal the structure–activity relationship of this series of compounds from the perspective of molecular electronic structure and verify the molecular mechanism of the previous herbicidal activity and enzyme inhibition experiments, we carried out density functional theory (DFT) calculations on the low-activity compound **I-09**, hit compound **I-20**, structurally optimized derivative **I-29** and commercial control agent penoxsulam. The key electronic structure parameters such as van der Waals surface electrostatic potential (ESP), frontier molecular orbitals (HOMO/LUMO), electronic chemical potential (μ), electrophilicity (ω) and dipole moment (D) are shown in Figure 8 and Table 4.

The van der Waals surface electrostatic potential distribution maps showed that the four compounds had highly similar electrostatic potential distribution patterns: the positive potential regions (red) were mainly concentrated at the heteroatom sites of the nitro-substituted benzene ring and the triazolopyrimidine ring, suggesting that these regions have a tendency to accept electrons and are prone to form hydrogen bonds or electrostatic interactions with electron-rich groups (e.g., -SH of cysteine and -NH_2_ of lysine) in the active center of the target enzyme (AHAS); the negative potential regions (blue) were distributed around the nitrogen and oxygen atoms of the triazolopyrimidine ring, indicating that these regions have electron-donating characteristics and can produce electrostatic attraction with electron-deficient sites (e.g., metal ions and positively charged amino acid residues) in the enzyme active site. This highly similar electrostatic potential distribution further confirms that this series of compounds has the same target binding mode as penoxsulam, with the AHAS enzyme as the core target, which is mutually corroborated with the conclusions of the previous in vivo enzyme inhibition experiments.

Based on the frontier molecular orbital theory, the HOMO-LUMO energy gap (Δ*E*) directly reflects the chemical reactivity of the molecule: a smaller energy gap means the molecule is more prone to electron transfer, usually corresponding to stronger biological activity. Although the energy gap of **I-20** (3.40 eV) was slightly smaller than that of **I-29** (3.63 eV), the lipophilicity of **I-29** (logP = 3.18) was closer to the herbicide development range (1.0~4.0), resulting in stronger membrane penetration ability. In addition, its higher electrophilicity (3.42 eV) and dipole moment (16.93 D) enhanced the interaction with the target enzyme, ultimately leading to better overall herbicidal activity.

Further analysis of the electronic characteristic parameters found that the electronic structure characteristics of **I-29** were directly related to its excellent biological activity: the absolute value of the electronic chemical potential of **I-29** (4.99 a.u.) was the highest among the four compounds, indicating that it has the weakest nucleophilicity, does not easily lose electrons, and is more conducive to maintaining stable binding with the target enzyme. In terms of electrophilicity (ω), **I-29** (3.42 eV) was significantly higher than penoxsulam (2.05 eV) and **I-09** (1.71 eV) and close to **I-20** (3.55 eV), indicating that it has a stronger electron-accepting ability and can form stronger interactions with electron-rich residues in the enzyme active center. In terms of dipole moment, **I-29** (16.93 D) had a relatively large molecular polarity, but the hydrophobic effect of the tert-butoxy group (logP = 3.18, within the development range) offset the negative impact of excessive molecular polarity on membrane penetration. At the same time, the increased dipole moment enabled the molecule to form stronger dipole–dipole interactions with polar amino acid residues (e.g., R377, S653) in the AHAS enzyme active center, which not only enhanced the stability of target binding but also synergistically improved the biological activity. The synergistic effect of these electronic characteristics made **I-29** perform better in target affinity and biological activity, which was completely consistent with the results of the herbicidal activity and enzyme inhibition experiments. These results indicate that the biological activity of the compounds is the synergistic effect of physicochemical properties, electronic structure and target binding ability, providing an important theoretical basis for the molecular design and optimization of novel AHAS inhibitors [31,32].

## 3. Materials and Methods

### 3.1. Equipment and Materials

All reagents and solvents used in this study were commercially purchased and used without further purification. Melting points of the compounds were determined using an uncorrected Cole-Parmer microscopic melting point apparatus. Nuclear magnetic resonance (NMR) spectra were recorded on a Bruker Avance III 500 spectrometer (Bruker BioSpin GmbH, Rudolf-Plank-Straße 23, 76275 Ettlingen, Germany) at a constant temperature of 25 °C, with tetramethylsilane (TMS) as the internal standard. High-resolution mass spectra (HRMS) were acquired on a Nexera UHPLC LC-30A (Shimadzu Corporation, Kyoto, Japan) tandem SCIEX X500B QTOF instrument (AB Sciex LLC, Framingham, MA, USA).

### 3.2. Synthesis

General Synthetic Procedure for Intermediate **2** [33]: 40 mmol of arylsulfonamide, 42 mmol of dimethyl cyanocarbonimidodithioate and 45 mmol of potassium hydroxide were dissolved in 300 mL of acetone, and the mixture was refluxed with stirring. The reaction was monitored by thin-layer chromatography (TLC) until the complete consumption of arylsulfonamide. After cooling, the mixture was filtered, and the filter cake was recrystallized from a DMSO–ethanol mixed solvent to afford pure compound **2** at a yield from 65% to 90%.

#### 3.2.1. General Synthetic Procedure for Intermediate **3**

A mixture of 5 mmol of compound **2**, 8 mmol of potassium hydroxide, 40 mL of 80% hydrazine hydrate and 20 mL of ethanol was refluxed with stirring for 12 h. A large amount of malodorous gas was generated during the reaction, the solution color gradually changed from colorless to yellow, and a large number of solids precipitated simultaneously. After the reaction, the mixture was filtered while hot, and the filter cake was washed with an appropriate amount of ethyl acetate three times and dried to obtain compound **3** with a yield from 70% to 85%, which was used directly in the next step without further purification.

#### 3.2.2. General Synthetic Procedure for Compound **I**

A mixture of 1 mmol of compound **3**, 1.5 mmol of ethyl acetoacetate or ethyl methoxyacetoacetate, and 40 mL of acetic acid was refluxed with stirring for 6 h. The solution color gradually changed from colorless to yellow during the reaction, the reaction system became clear gradually with heating, and then solids precipitated slowly. After cooling, the mixture was filtered, and the solids precipitated from the concentrated filtrate were washed with an appropriate amount of ethyl acetate three times and combined with the aforementioned filter cake. The combined solids were recrystallized from a DMSO/ethanol mixed solvent (*v*/*v* = 1:4–1:6) to afford compound **I** with a yield from 40% to 95%.

#### 3.2.3. Synthetic Procedure for Compound **I-29**

An amount of 2.7 mmol of compound **I-20** was suspended in 15 mL of phosphoryl chloride under an ice–salt bath (<−5 °C). The mixture was slowly warmed to dissolve the solids with stirring and then further heated to reflux for 2 h. After cooling, the mixture was filtered to obtain a dark red solid. The filter cake was dispersed in dichloromethane, and saturated sodium bicarbonate solution was added with vigorous stirring until no more gas bubbles were generated. The mixture was filtered and dried to afford a dark red solid, which was then dissolved in 10 mL of DMSO and cooled in an ice bath. Then, 4 mL of 10% potassium *tert*-butoxide in *tert*-butanol was added dropwise with vigorous stirring. After the dropwise addition, the mixture was heated to 80 °C and reacted for 4 h. After cooling, a saturated ammonium chloride solution was added to quench the reaction, and the pH of the system was adjusted to 5~6 with 0.5 M hydrochloric acid in an ice bath. The mixture was extracted with dichloromethane (3 × 15 mL), and the organic phases were combined and concentrated under reduced pressure. The residue was purified by column chromatography with gradient elution (ethyl acetate–petroleum ether, *v*/*v* = 1:5–2:1) to afford compound **I-29** with a yield of 35%.

The corresponding physicochemical properties and structural characterization data (^1^H, ^13^C NMR spectral data, HRMS and melting points) of the target compounds are listed in the Supporting Information.

### 3.3. Single-Crystal Diffraction of ***I-09***

Compound **I-09** was dissolved in a mixed solvent of dichloromethane and methanol, and single crystals of **I-09** were obtained by slow volatilization of the solvent at room temperature. Single-crystal X-ray diffraction (SC-XRD) was performed on a Bruker D8 VENTURE diffractometer (Bruker AXS GmbH, Karlsruhe, Germany), and data analysis was carried out using Olex-2 1.5 software. Relevant information is provided in the Supporting Information (Tables S2 and S3).

### 3.4. Herbicidal Activity Assay

#### 3.4.1. Preliminary Screening

The assay was conducted in accordance with the Pesticides Guidelines for Laboratory Bioactivity Tests—Part 4: Foliar Spray Application Test for Herbicide Activity (NY/T 1155.4-2006) [34], as mandated by the industry standards of the People’s Republic of China. Test compounds were initially dissolved in dimethyl sulfoxide (DMSO) containing 1% Tween-80 emulsifier to create a stock solution at a concentration of 10 mg/mL. This stock solution was subsequently serially diluted with an aqueous solution containing 1% Tween-80 to produce test solutions with concentrations of 0.311, 0.133, and 0.067 mg/mL, which corresponded to active ingredient dosages of 70, 30, and 15 g a.i./ha, respectively, after applying 10 mL of the test solution to each 8 cm × 8 cm test pot. Three common weeds—*Echinochloa crus-galli*, *Sorghum cannabinum*, and *Amaranthus theophrasti*—were selected as target species for the preliminary screening of herbicidal activity. The weeds were cultivated in a constant temperature incubator, and once they reached the 2–3 leaf stage, the test solutions were applied using a nebulizer to evaluate post-emergence herbicidal activity. Penoxsulam was treated under the same conditions and served as the positive control. Each experiment was repeated three times. Fourteen days post-treatment, the growth of the weeds was visually assessed, and the levels of herbicidal activity for both the test compounds and the positive control were evaluated based on the observed symptoms of weed injury.

#### 3.4.2. Rescreening

The assay method was the same as that of the preliminary screening, with the active ingredient dosages set at 30, 15 and 7.5 g a.i./ha. Six common weeds, *E. crusgalli*, *S. cannabina*, *Eleusine indica*, *A. theophrasti*, *Eclipta prostrata* and *Digitaria sanguinalis*, were selected as the target weeds, and the assay was conducted when the weeds grew to the 2~3 leaf stage in a constant temperature incubator.

The activity rating scale used is denoted as follows: “+++++”—almost all plants were completely chlorotic and necrotic from roots to aerial parts or tissue was lignified, accompanied by lodging, without any growth potential, and with only a small amount of inactive residues remaining; “++++”—the main parts of most plants (stems and functional leaves) were chlorotic, withered or the tissue was lignified, some plants were necrotic or lodged, and only a small part of the plant roots retained weak growth potential without obvious recovery ability; “+++”—the main parts of about half of the plants showed chlorosis, withering or tissue lignification, some plants exhibited lodging, and the remaining plants still retained certain growth potential but with significant growth inhibition and no normal development ability; “++”—a small number of plants only showed chlorosis at the seedling growing points or new leaves, no chlorosis, withering, lignification or necrosis in the main plant parts, no lodging of the whole plant, and were still with certain growth potential and had mild growth inhibition; “+”—the main parts of almost all plants developed normally, only individual plants showed slight wilting at the seedling growing points, and there were no injury symptoms such as chlorosis and withering, with good plant growth potential and a rapidly recoverable inhibitory effect; “-”—the plant growth status was not significantly different from the blank control, was without any injury symptoms, was with well-developed root systems and robust stems and leaves, and showed normal growth and development and no inhibitory effect.

### 3.5. In Vivo AHAS Inhibition Assay [35] Determination of AHAS Inhibition Activity

*E. crusgalli* seeds were soaked in water to accelerate germination until the roots reached 1–1.5 cm in length. In a Petri dish with two pieces of filter paper, 5 mL of the test solution (concentration gradients: 50, 25, 12.5, 6.25 and 3.125 μM) was added, followed by 0.5 g of pregerminated *E. crusgalli* roots. The dish was incubated in the dark at 29 ± 1 °C for 48 h. After incubation, all samples were collected and fully ground with phosphate-buffered saline (PBS). The mixture was centrifuged at 4000 r/min for 15 min to separate the supernatant. Subsequently, the samples were processed using an ELISA kit, and the AHAS enzyme activity was quantitatively analyzed based on the absorbance at 425 nm measured with a microplate reader.

All regression equations, IC_50_ values, standard errors (SE) and R^2^ coefficients were generated using GraphPad Prism 9: data were imported as an XY table (X = agent concentration, Y = AHAS inhibition rate) and analysis was performed using the Dose–Response (Inhibition) module with the fitting mode of “log(inhibitor) vs. response—Variable slope (four-parameter logistic model)”. The “Linear Regression Equation” label in the table refers to the simplified presentation of the four-parameter logistic nonlinear regression equation generated by GraphPad Prism 9 (free trial).

Statistical Analysis. Experimental data were processed using the four-parameter logistic nonlinear regression model in GraphPad Prism 10 software. The IC_50_ value represents the herbicide concentration at which 50% of AHAS enzyme activity is inhibited, calculated using Equation (1):

*Y* = *C* + (*D* − *C*)/[1 + (*X*/IC_50_)*^b^*] (1)

where *Y* is the ratio of AHAS catalytic activity in each treatment to that in the control; *X* is the herbicide concentration (μM); *C* and *D* are the lower and upper limits, respectively; and parameter *b* is the slope.

### 3.6. Molecular Docking [22]

Using the coordinates of penoxsulam in the single crystal structure of the *At*AHAS–penoxsulam complex as the center [15], the binding site of AHAS herbicides was identified as the active pocket [202:A,352:A,377:A,574:A]. The small-molecule structures of the hit and lead compounds were drawn using Marvin Sketch (Version 25.1.3 developed by ChemAxon, http://www.chemaxon.com) and converted into SMILES strings, which were then transformed into 3D structures using the RdKit software (version 2025.09.6, https://www.rdkit.org), followed by hydrogenation, charge assignment and energy minimization. Both the receptor and ligand small molecules were converted into standard pdbqt files using ADT of mgltools. The coordinates of the active pocket binding site were 63.05, −75.23, and −46.54 (Å), and the size of the docking box was 30 Å × 30 Å × 30 Å. Autodock Vina was used to dock the target compounds into the binding site of AHAS, and the visualization of graphics was completed using PyMOL 2.0 software [26].

### 3.7. Density Functional Theory (DFT) Calculation

The structures of representative compounds were constructed and optimized. DFT calculations were performed in Gaussian 16, and the results were processed and analyzed using GaussView 6.0. The single-electron energies of the frontier molecular orbitals (E_HOMO/E_LUMO) were used to determine the electronic chemical potential (*μ*) and chemical hardness (*η*) of the representative compounds using Equations (2) and (3):

(2) $$\mu =\frac{E_{HOMO}+E_{LUMO}}{2}$$

(3) $${\eta =E}_{LUMO}-E_{HOMO}$$

The global electrophilicity index (*ω*) was calculated using the electronic chemical potential (*μ*) and chemical hardness (*η*) according to Equation (4):

(4) $$\omega =\frac{\mu^{2}}{2\eta }$$

## 4. Summary and Future Work

Given the metabolic detoxification associated with the 5-demethylation of pyroxsulam, this study aimed to replace the 5-methoxy group on the triazolopyrimidine ring with various alkyl groups. Consequently, a series of *N*-(7-oxo-4,7-dihydro-[1,2,4]triazolo[1,5-a]pyrimidin-2-yl)arylsulfonamides was synthesized and displayed substantial structural diversification potential. This synthetic strategy involved initially constructing the triazole ring, followed by the formation of the triazolopyrimidine skeleton using sulfonamide as the starting material, from which the herbicidal hit compound **I-20** was identified. The herbicidal activity displayed by this class of compounds suggests that the 7-demethylation metabolic pathway of pyroxsulam may not function as its detoxification pathway. However, the weak lipophilicity of these compounds can hinder their herbicidal efficacy. To address this issue, we further converted the 7-carbonyl group into a tert-butoxy group, leading to the discovery of the new lead compound **I-29**, which exhibited herbicidal activity comparable to, or even surpassing, that of penoxsulam against the tested weeds. Systematically studies revealed that **I-29** demonstrated a receptor binding mode and herbicidal activity phenotype highly consistent with penoxsulam, and its spatial structure appeared even more favorable for inhibiting FAD-mediated AHAS activity. The current research not only enhances the understanding of the 5-demethylation metabolic resistance of triazolopyrimidine sulfonamide herbicides but also offers new perspectives for the further development of AHAS-inhibiting triazolopyrimidine sulfonamide herbicides [36]. Building on the findings of this work, we will further perform molecular optimization and in-depth studies using this established method in subsequent research, with the aim of identifying candidate molecules with greater potential for commercial development as herbicides.

## Figures and Tables

**Table 1:** Preliminary herbicidal activity of title compounds ^a^.

Compound	*E.C.* ^b^ (g a.i./ha)			*S.C.* ^b^ (g a.i./ha)			*A.T.* ^b^ (g a.i./ha)		
	70	30	15	70	30	15	70	30	15
**I-01**	+	+	+	+++	++	+	++++	+	-
**I-02**	+	+	-	++++	++++	-	+++++	+	-
**I-03**	+	+	-	++++	+++	-	+++++	+	-
**I-04**	++++	+	-	++++	+++	-	+++++	+++++	++++
**I-05**	+	+	-	+++++	+++++	-	+++++	+	-
**I-06**	+	+++	-	++++	+	-	+++++	+	-
**I-07**	++	+	-	+++++	++++	-	+++++	++++	++
**I-08**	++	+++	-	++++	++	-	+++++	+	-
**I-09**	+	++	+++	+++++	++++	+++	+++++	+++++	+++
**I-10**	+	+	-	+++++	++++	-	+++++	++	+
**I-11**	+	+	-	+++++	++++	-	+++++	+	-
**I-12**	++++	+	-	++++	+++	-	+++++	++	+
**I-13**	+	++	-	+++++	++++	-	+++++	+	-
**I-14**	+++	+	-	++++	+++	-	+++++	++	-
**I-15**	++++	+	-	+++++	+++++	-	+++++	+	-
**I-16**	++	+++	++	++++	+++	++	+++++	+	-
**I-17**	++++	++++	-	++++	+++	-	+++++	+	-
**I-18**	++	++	-	+++	+++	+	+++++	++++	++
**I-19**	++	+	-	++++	++++	+	++++	+	-
**I-20**	+++++	+++++	+++	+++++	+++++	+++++	+++++	+++++	+++++
**I-21**	++++	++++	-	+++++	+++++	++	+++++	+	-
**I-22**	+	++++	-	++++	++	-	+++++	+	-
**I-23**	++	+	-	+++	+	-	+++++	+	+
**I-24**	++++	++++	-	+++++	++++	-	+++++	+	+
**I-25**	++++	++++	+	+++++	++++	-	+++++	+	-
**I-26**	++	-	-	+++++	+++++	-	+++++	+	-
**I-27**	++	++++	-	+++++	++++	-	+++++	+	-
**I-28**	++	+	-	+++++	+++++	-	+++++	+	+
penoxsulam	+++++	+++++	+++++	+++++	+++++	+++++	+++++	+++++	+++++

^a^. Fourteen days after treatment, the herbicidal activity of each compound at different dosages was determined using a scoring scale of 0 to 100%. Rating scale for the growth inhibition percentage in relation to the untreated control: +++++, ≥90%; ++++, ≥70%; +++, ≥50%; ++, ≥30%; +, ≥10%; -, <10%. ^b^. Abbreviations: *E.C.* = *E. crusgalli*, *S.C.* = *S. cannabina*, *A.T.* = *A. theophrasti*.

**Table 2:** Comparison of herbicidal activity of compounds **I-20**, **I-29** and penoxsulam ^a^.

Compound	Dosage (g a.i./ha)	*E.C.* ^b^	*S.C.* ^b^	*E.I.* ^b^	*A.T.* ^b^	*E.P.* ^b^	*D.S.* ^b^
**I-20**	30	+++++	+++++	++++	+++++	+++++	+
**I-29**		+++++	+++++	+++++	+++++	+++++	+++++
Penoxsulam		+++++	+++++	+++++	+++++	+++++	+++++
**I-20**	15	+++	+++++	++	+++++	+++++	-
**I-29**		+++++	+++++	+++++	+++++	+++++	++++
Penoxsulam		+++++	+++++	++++	+++++	+++++	+
**I-20**	7.5	++	++	+	++++	++	-
**I-29**		++++	+++++	+++	+++++	++++	+++
Penoxsulam		++++	+++++	++	+++++	+++	+

^a^. The herbicidal inhibitory rate was determined fourteen days after treatment by 0 to 100% with each dosage of compound. Rating scale for the growth inhibition percentage in relation to the untreated control: +++++, ≥90%; ++++, ≥70%; +++, ≥50%; ++, ≥30%; +, ≥10%; -, <10%. ^b^. Abbreviations: *E.C.* = *E. crusgalli*, *S.C.* = *S. cannabina*, *E.I.* = *E. indica*, *A.T.* = *A. theophrasti*, *E.P.* = *E. prostrata*, *D.S.* = *D. sanguinalis*.

**Table 3:** In vivo AHAS inhibitory activity in *E. crusgalli* (IC_50_, μM).

Compound	Linear Regression Equation	IC_50_ ± SE (μM)	R^2^
**I-09**	y = 17.52 + (82.48)/[1 + (x/11.88)^1.23^]	11.88 ± 0.096	0.9995
**I-20**	y = 41.54 + (58.46)/[1 + (x/9.21)^1.15^]	9.213 ± 0.079	0.9990
**I-29**	y = 35.00 + (65.00)/[1 + (x/8.09)^1.08^]	8.088 ± 0.087	0.9992
penoxsulam	y = 32.10 + (67.90)/[1 + (x/8.94)^1.12^]	8.939 ± 0.081	0.9995

**Table 4:** HOMO-LUMO energy levels, energy gaps and related physicochemical parameters of **I-09**, **I-20**, **I-29** and penoxsulam.

Compound	*E*_HOMO_ (eV)	*E*_LUMO_ (eV)	Δ*E_gap_* (eV)	*µ* (a.u)	*ω* (eV)	Dipole (D)
**I-09**	−6.6	−1.64	4.96	4.12	1.71	13.34
**I-20**	−6.61	−3.21	3.4	4.91	3.55	5.43
**I-29**	−6.8	−3.17	3.63	4.99	3.42	16.93
Penoxsulam	−6.87	−2.04	4.83	4.46	2.05	10.11

## Data Availability

Data is contained within the article or Supplementary Material.

## Supplementary Materials

The following supporting information can be downloaded at https://www.mdpi.com/article/10.3390/molecules31061008/s1, ^1^H NMR, ^13^C NMR, and HRMS spectrum data of target compounds (PDF); Ref. [37] are cited in the Supplementary Materials.
