# Supplementary material for: Synthesis and Herbicidal Activity of Novel N-(7-Oxo-4,7-dihydro-[1,2,4]triazolo[1,5-a]pyrimidin-2-yl)arylsulfonamides
Source: Molecules. 2026 Mar 17;31(6):1008. doi: 10.3390/molecules31061008 (PMC13029049; doi:10.3390/molecules31061008)
Supplement: Supplementary file 1 [file molecules-31-01008-s001.zip › molecules-4201275-supplementary.pdf]

Synthesis and Herbicidal Activity of Novel N-7-oxo-4,7-dihydro-[1,2,4]triazolo-[1,5-a]pyrimidin-2-yl)arylsulfonamides

Xun Li[1], Yiyi Tian[1], Xianjun Tang[1], Jia-Qi Li*,[1], Huizhe Lu[1], Xiuhai Gan[2], Yumei Xiao[1], and Zhaohai Qin*,[1]

[1]College of Science, China Agricultural University, Beijing 100193, China

[2]State Key Laboratory of Green Pesticide, Key Laboratory of Green Pesticide and Agricultural Bioengineering, Ministry of Education, Guizhou University, Guiyang 550025, China

* Corresponding author: Tel.(Fax): 86-10-62732958; E-mail: luhz@cau.edu.cn(LH) ; qinzhaohai@263.net(ZQ)

Supporting Information

1. Physico-chemical properties and structural characterization data of target compounds

Data for compound **I-01. N-(5-methyl-7-oxo-1,7-dihydro-[1,2,4]triazolo[1,5-a]pyrimidin-2-yl)benzenesulfonamide.** White solid, m.p. 237–238 °C, yield 89%. ^{1}H NMR (500 MHz, DMSO-d$_6$) δ8.11 – 7.77 (m, 4H), 7.57 (d, J = 8.0 Hz, 1H), 5.76 (s, 1H), 2.23 (s, 3H). ^{13}C NMR (126 MHz, DMSO-d$_6$) δ 155.58, 150.48, 136.13, 131.83, 129.84, 126.45, 122.24, 99.46, 21.53. HRMS(ESI) calcd. for $C_{12}H_{11}N_5O_3S$: $[M]^+$ 305.0583, found 305.0622.

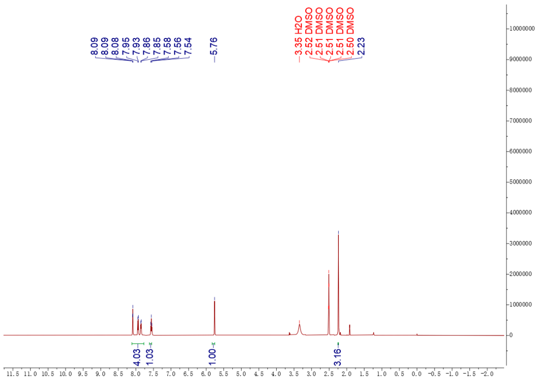

^{1}H NMR of compound **I-01**

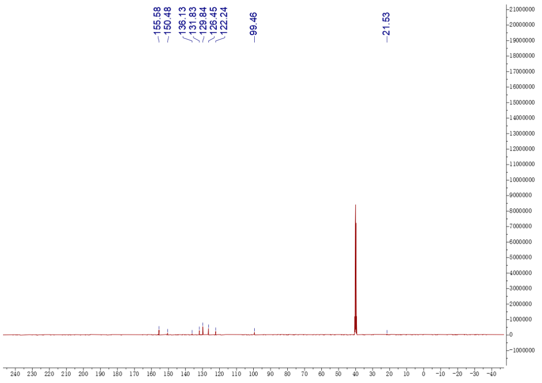

^{13}C NMR of compound **I-01**

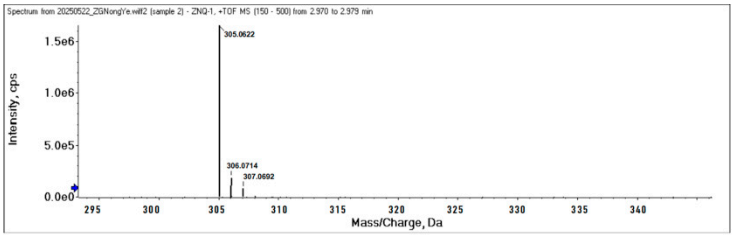

HRMS of compound **I-01**

Data for compound **I-02. 2-methyl-N-(5-methyl-7-oxo-1,7-dihydro-[1,2,4]triazolo[1,5-a]pyrimidin-2-yl)benzenesulfonamide**. White solid, m.p. 240–241°C, yield 92%. ^{1}H NMR (500 MHz, DMSO-d$_6$) δ 13.14 (s, 1H), 12.20 (s, 1H), 8.15 – 8.10 (m, 1H), 7.70 – 7.63 (m, 2H), 7.58 (ddd, J = 8.4, 5.9, 2.6 Hz, 1H), 5.75 (s, 1H), 2.22 (s, 3H), 1.91 (s, 3H). ^{13}C NMR (126 MHz, DMSO-d$_6$) δ 172.49, 156.16, 155.55, 150.85, 150.47, 137.77, 135.09, 132.19, 132.15, 131.19, 128.05, 99.29, 21.54, 18.90. HRMS(ESI) calcd. for $C_{13}H_{14}N_5O_3S$: $[M+H]^+$ 320.0812, found 320.0808.

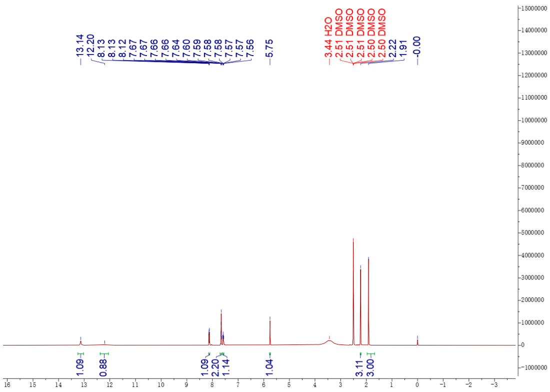

^{1}H NMR of compound **I-02**

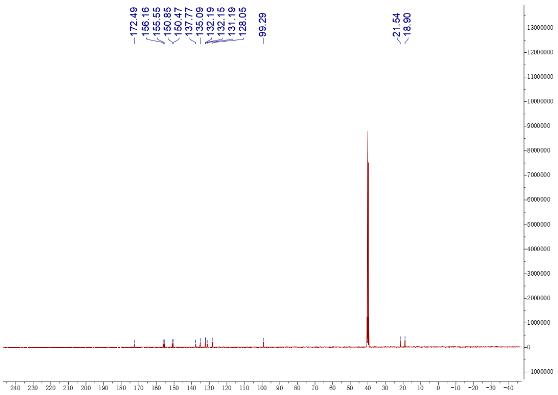

^{13}C NMR of compound **I-02**

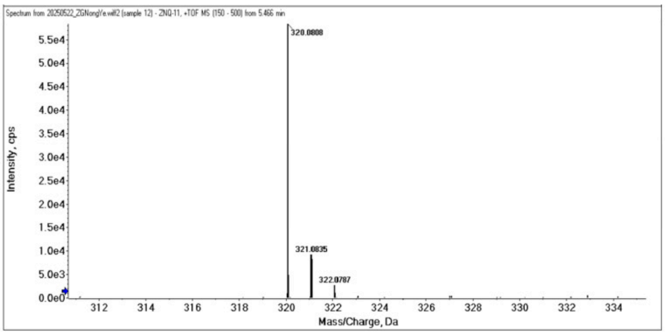

HRMS of compound **I-02**

Data for **I-03. 4-methyl-N-(5-methyl-7-oxo-1,7-dihydro-[1,2,4]triazolo[1,5-a]pyrimidin-2-yl)benzenesulfonamide**. White solid, m.p.241–242 °C, yield 95%. ^{1}H NMR (500 MHz, DMSO-d$_6$) δ 8.11 – 7.77 (m, 4H), 7.57 (d, J = 8.0 Hz, 1H), 5.76 (s, 1H), 2.23 (s, 3H).^{13}C NMR (126 MHz, DMSO) δ 155.58, 150.48, 136.13, 131.83, 129.84, 126.45, 122.24, 99.46, 21.53. HRMS(ESI) calcd. for $C_{13}H_{14}N_5O_3S$: $[M+H]^+$ 320.0812, found 320.0808.

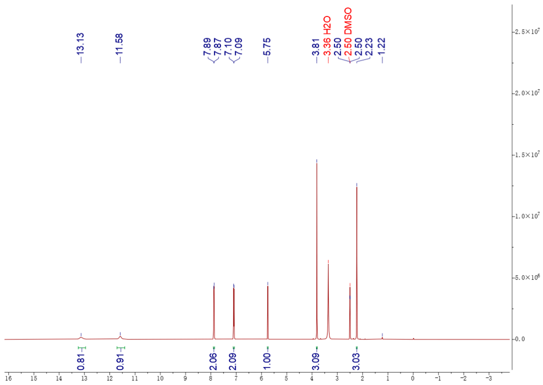

^{1}H NMR of compound **I-03**

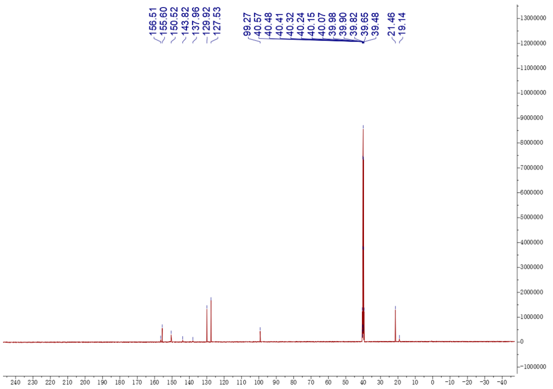

^{13}C NMR of compound **I-03**

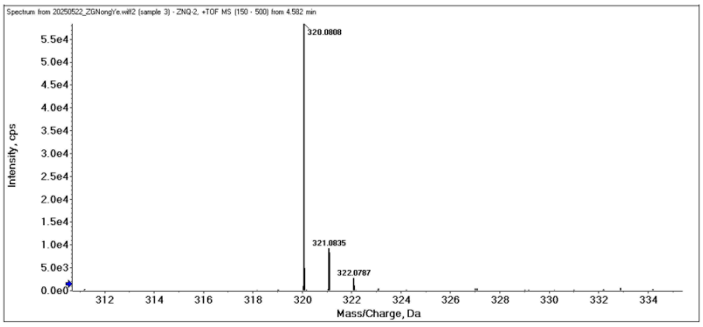

HRMS of compound **I-03**

Data for **I-04. 2-chloro-N-(5-methyl-7-oxo-1,7-dihydro-[1,2,4]triazolo[1,5-a]pyrimidin-2-yl)benzenesulfonamide.** White solid, m.p. 255–256°C, yield 85%. ^{1}H NMR (500 MHz, DMSO-d_6) δ 13.12 (s, 1H), 12.19 (s, 1H), 8.15 – 8.10 (m, 1H), 7.70 – 7.62 (m, 2H), 7.58 (ddd, J = 8.4, 6.1, 2.6 Hz, 1H), 5.75 (s, 1H), 1.91 (s, 3H). ^{13}C NMR (126 MHz, DMSO-d_6) δ 156.17, 155.55, 150.82, 150.46, 139.51, 135.72, 134.98, 132.31, 128.53, 119.60, 99.30, 18.91. HRMS(ESI) calcd. for $C_{12}H_{11}ClN_5O_3S$: $[M+H]^+$ 340.0266, found 340.0261.

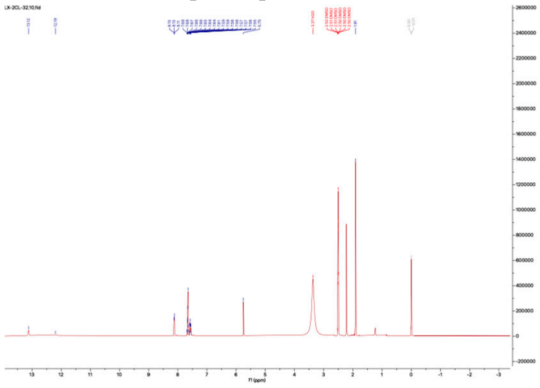

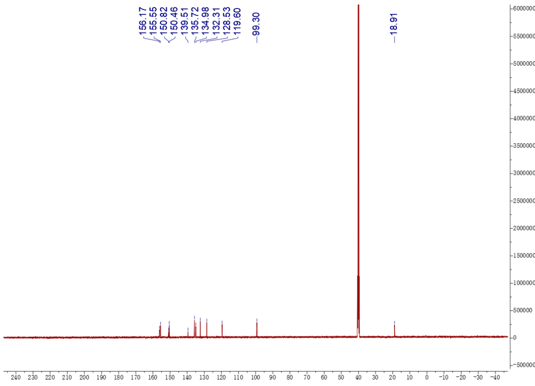

^{1}H NMR of compound **I-04** ^{13}C NMR of compound **I-04**

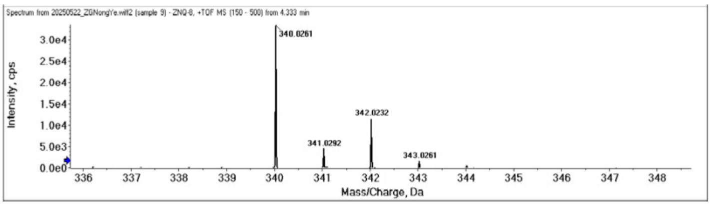

HRMS of compound **I-04**

Data for **I-05. 4-chloro-N-(5-methyl-7-oxo-1,7-dihydro-[1,2,4]triazolo[1,5-a]pyrimidin-2-yl)benzenesulfonamide.**[1] White solid, m.p.245–246 °C, yield 90%. ^{1}H NMR (500 MHz, DMSO-d_6) δ 13.16 (s, 1H), 11.90 (s, 1H), 7.99 – 7.93 (m, 2H), 7.73 – 7.66 (m, 2H), 5.79 – 5.76 (m, 1H), 2.24 (s, 3H). ^{13}C NMR (126 MHz, DMSO-d_6) δ 156.31, 155.55, 150.87, 150.56, 139.44, 138.49,

129.78, 129.48, 99.32, 18.94. HRMS(ESI) calcd. for $C_{12}H_{11}ClN_5O_3S$: $[M+H]^+$ 340.0266, found 340.0264

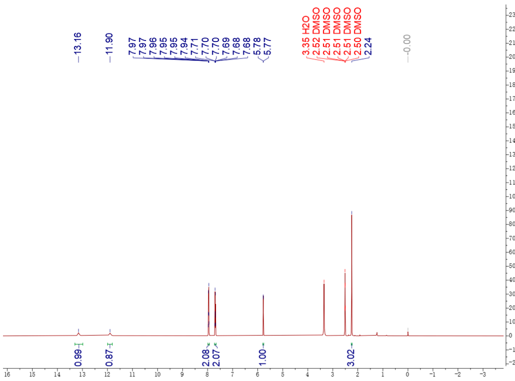

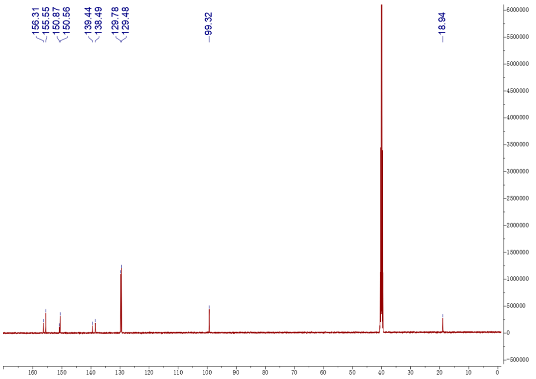

^{1}H NMR of compound **I-05** ^{13}C NMR of compound **I-05**

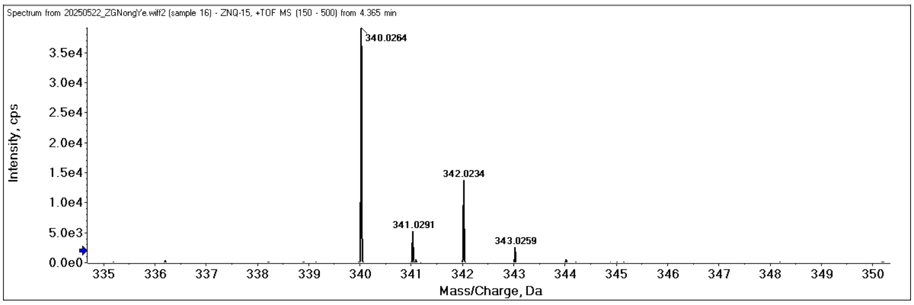

HRMS of compound **I-05**

Data for **I-06. 2-fluoro-N-(5-methyl-7-oxo-1,7-dihydro-[1,2,4]triazolo[1,5-a]pyrimidin-2-yl)benzenesulfonamide.**White solid, m.p.244–245 °C, yield 80%. ^{1}H NMR (500 MHz, DMSO-d_6) δ 13.22 (s, 1H), 11.85 (s, 1H), 8.06 – 7.99 (m, 2H), 7.46 (t, J = 8.8 Hz, 2H), 5.77 (s, 1H), 2.25 (s, 3H). ^{13}C NMR (126 MHz, DMSO-d_6) δ 172.46, 165.96, 163.95, 156.39, 155.56, 150.90, 150.55, 136.95, 136.93, 130.69, 130.62, 116.90, 116.71, 99.29, 21.55, 18.93, 11.67. HRMS(ESI) calcd. for $C_{13}H_{13}FN_5O_4S$: $[M+H]^+$ 324.0561, found 324.0582.

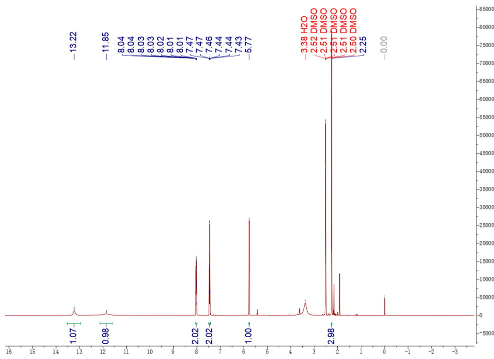

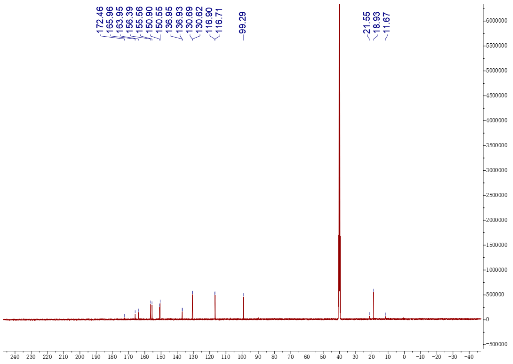

^{1}H NMR of compound **I-06** ^{13}C NMR of compound **I-06**

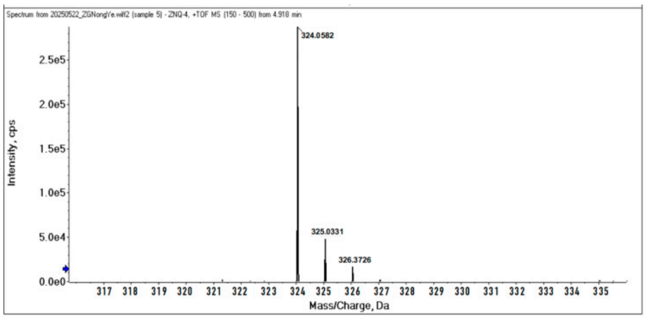

HRMS of compound **I-06**

Data for **I-07. 3-fluoro-N-(5-methyl-7-oxo-1,7-dihydro-[1,2,4]triazolo[1,5-a]pyrimidin-2-yl)benzenesulfonamide**. White solid, m.p.240–241 °C, yield 81%. ^{1}H NMR (500 MHz, DMSO-d_6) δ 13.16 (s, 1H), 11.94 (s, 1H), 7.82 – 7.73 (m, 2H), 7.68 (td, J = 8.1, 5.4 Hz, 1H), 7.55 (td, J = 8.6, 2.6 Hz, 1H), 5.78 (s, 1H), 2.24 (s, 3H). ^{13}C NMR (126 MHz, DMSO-d_6) δ 162.91, 160.93, 155.57, 150.56, 132.07, 123.69, 123.67, 120.89, 114.75, 114.56, 99.35, 18.97. HRMS(ESI) calcd. for $C_{12}H_{11}FN_5O_3S$: $[M+H]^+$ 324.0561, found 324.0563.

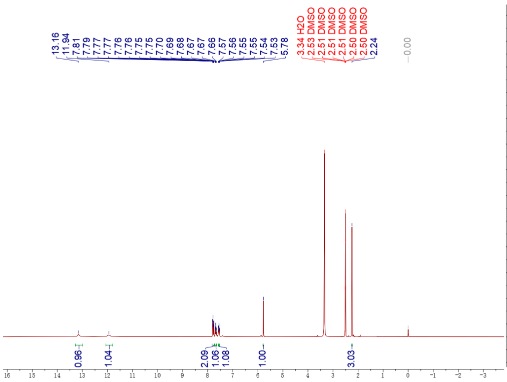

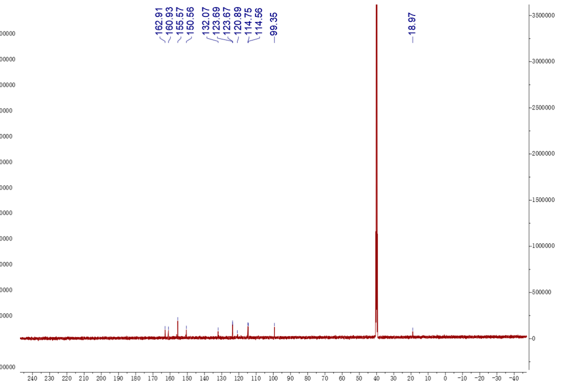

^{1}H NMR of compound **I-07** ^{13}C NMR of compound **I-07**

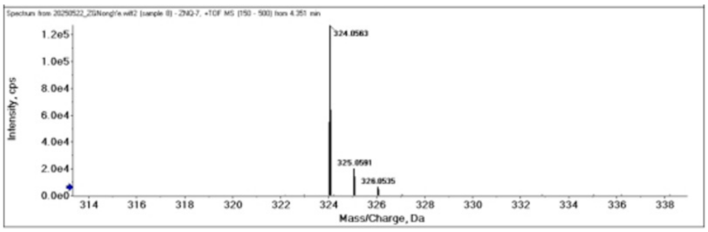

HRMS of compound **I-07**

Data for **I-08. 4-fluoro-N-(5-methyl-7-oxo-1,7-dihydro-[1,2,4]triazolo[1,5-a]pyrimidin-2-yl)benzenesulfonamide**. White solid, m.p. 237–238°C, yield 90%. ^{1}H NMR (500 MHz, DMSO) δ 13.16 (s, 1H), 11.90 (s, 1H), 7.97 – 7.94 (m, 2H), 7.72 – 7.66 (m, 2H), 5.79 – 5.76 (m, 1H), 2.24 (s, 3H). ^{13}C NMR (126 MHz, DMSO) δ 156.31, 155.55, 150.87, 150.56, 139.44, 138.49, 129.78, 129.48, 99.32, 18.94. HRMS(ESI) calcd. for $C_{12}H_{11}FN_5O_3S$: $[M+H]^+$ 324.0561, found 324.0563.

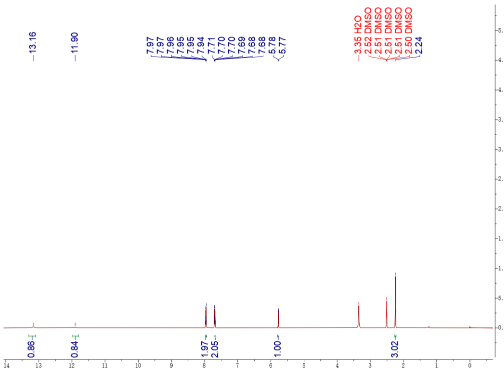

^{1}H NMR of compound **I-08**

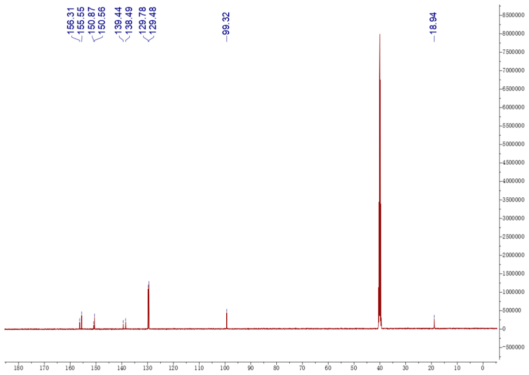

^{13}C NMR of compound **I-08**

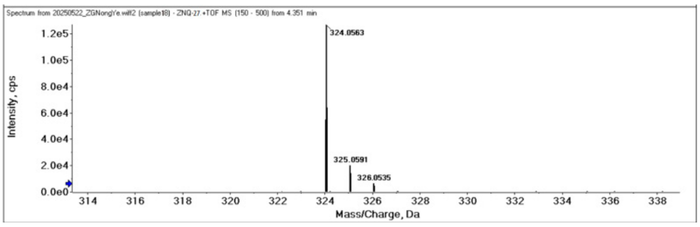

HRMS of compound **I-08**

Data for **I-09. 2-bromo-N-(5-methyl-7-oxo-1,7-dihydro-[1,2,4]triazolo[1,5-a]pyrimidin-2-yl)benzenesulfonamide.**White solid, m.p.267–268 °C, yield 70%. ^{1}H NMR (500 MHz, DMSO-d_6) δ 13.11 (s, 1H), 12.22 (s, 1H), 8.15 (dd, J = 7.9, 1.7 Hz, 1H), 7.84 (dd, J = 7.9, 1.3 Hz, 1H), 7.62 (td, J = 7.7, 1.3 Hz, 1H), 7.56 (td, J = 7.6, 1.8 Hz, 1H), 5.76 (s, 1H), 2.22 (s, 3H). ^{13}C NMR (126 MHz, DMSO-d_6) δ 154.26, 153.63, 148.55, 137.60, 133.80, 133.06, 130.40, 126.62, 117.69, 97.39, 17.00. HRMS(ESI) calcd. for $C_{12}H_{11}BrN_5O_3S$: $[M+H]^+$ 383.9760, found 383.9761.

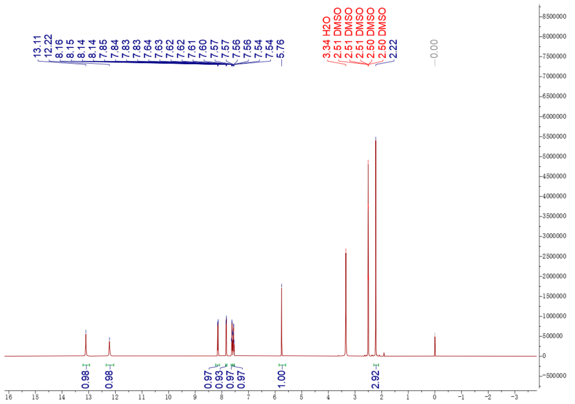

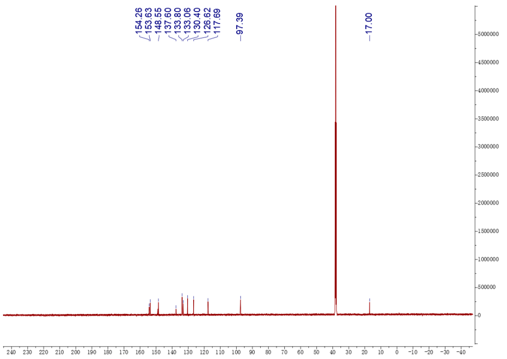

^{1}H NMR of compound **I-09**

^{13}C NMR of compound **I-09**

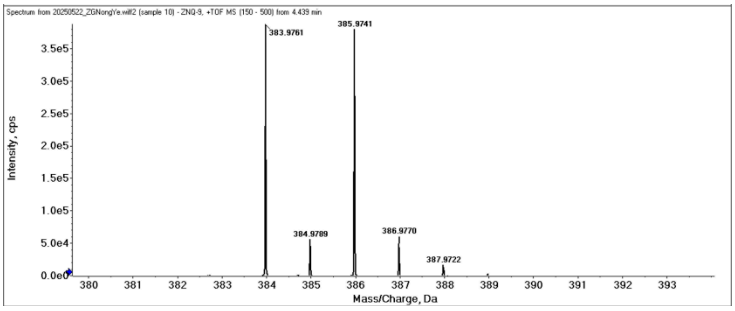

HRMS of compound **I-09**

Data for **I-10**. **3-cyano-N-(5-methyl-7-oxo-1,7-dihydro-[1,2,4]triazolo[1,5-a]pyrimidin-2-yl)benzenesulfonamide.** Yellow solid, m.p.238–239 °C, yield 65%. ^{1}H NMR (500 MHz, DMSO-d_6) δ 13.16 (s, 1H), 12.09 (s, 1H), 8.36 (t, J = 1.8 Hz, 1H), 8.25 (dt, J = 8.1, 1.5 Hz, 1H), 8.16 (dt, J = 7.8, 1.4 Hz, 1H), 7.84 (t, J = 7.9 Hz, 1H), 5.78 (s, 1H), 2.24 (s, 3H). ^{13}C NMR (126 MHz, DMSO-d_6) δ 156.08, 156.07, 155.53, 150.59, 141.82, 137.26, 131.96, 131.25, 131.19, 117.94, 112.78, 99.38, 18.97. HRMS(ESI) calcd. for $C_{13}H_{11}N_6O_3S$: $[M+H]^+$ 331.0608, found 331.0607.

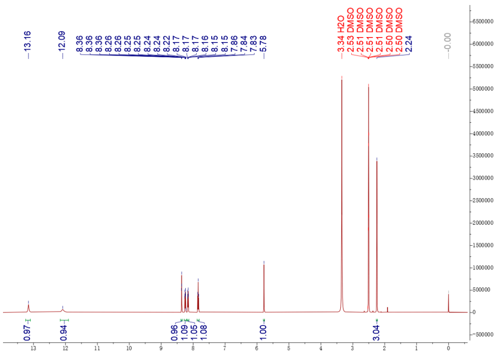

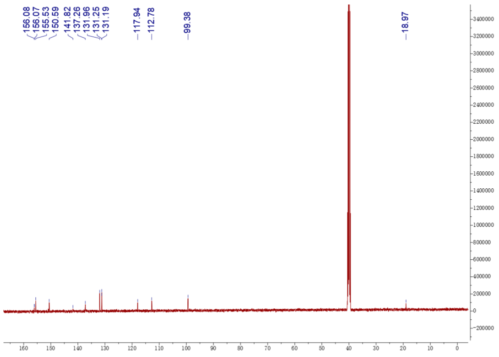

^{1}H NMR of compound **I-10**

^{13}C NMR of compound **I-10**

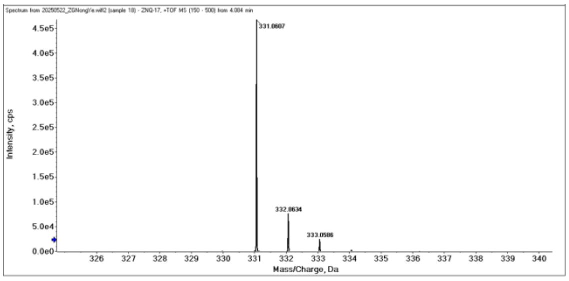

HRMS of compound **I-10**

Data for **I-11. N-(5-methyl-7-oxo-1,7-dihydro-[1,2,4]triazolo[1,5-a]pyrimidin-2-yl)-2-(trifluoromethyl)benzenesulfonamide.** White solid, m.p.210–211℃, yield 85%. ^{1}H NMR (500 MHz, DMSO-d_6) δ 13.22 (s, 1H), 12.25 (s, 1H), 8.30 – 8.25 (m, 1H), 8.01 (dd, J = 7.7, 1.6 Hz, 1H), 7.95 – 7.84 (m, 2H), 5.78 (s, 1H), 2.24 (s, 3H). ^{13}C NMR (126 MHz, DMSO-d_6) δ 156.25, 155.57, 150.98, 150.52, 139.16, 133.99, 133.68, 131.75, 128.90, 128.85, 126.71, 126.45, 124.37, 122.19, 120.02, 99.31, 18.93. HRMS(ESI) calcd. for $C_{13}H_{11}F_3N_5O_3S$: $[M+H]^+$ 374.0529, found 374.0527.

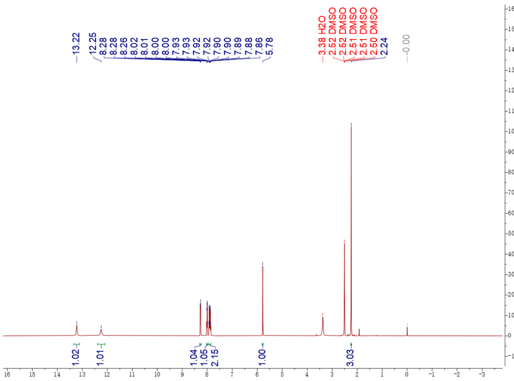

^{1}H NMR of compound **I-11**

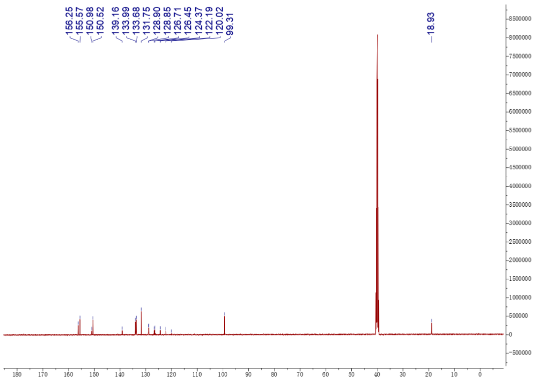

^{13}C NMR of compound **I-11**

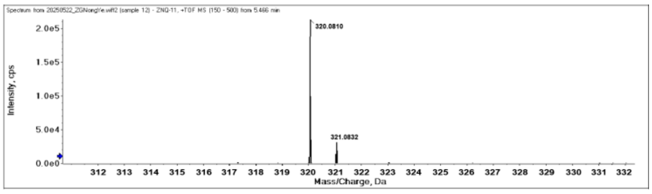

HRMS of compound **I-11**

Data for **I-12. N-(5-methyl-7-oxo-1,7-dihydro-[1,2,4]triazolo[1,5-a]pyrimidin-2-yl)-4-(trifluoromethyl)benzenesulfonamide**. White solid, m.p.202–203 ℃, yield 90%. ^{1}H NMR (500 MHz, DMSO-d_6) δ 13.21 (s, 1H), 12.24 (s, 1H), 8.27 (d, J = 7.7 Hz, 1H), 8.03 – 7.98 (m, 1H), 7.95

– 7.84 (m, 2H), 5.78 (s, 1H), 2.24 (s, 3H). ^{13}C NMR (126 MHz, DMSO-d_6) δ 156.25, 155.58, 150.52, 139.17, 133.68, 131.75, 128.90, 128.85, 126.97, 126.71, 126.45, 126.19, 124.37, 99.31, 18.93. HRMS(ESI) calcd. for $C_{13}H_{11}F_3N_5O_3S$: $[M+H]^+$ 374.0529, found 374.0524.

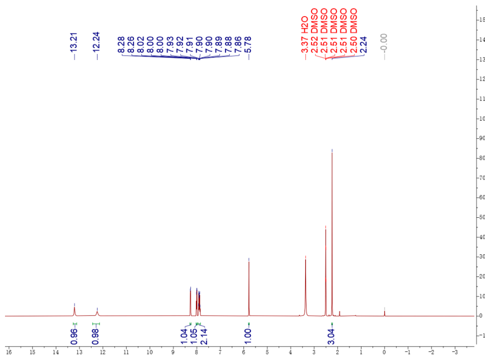

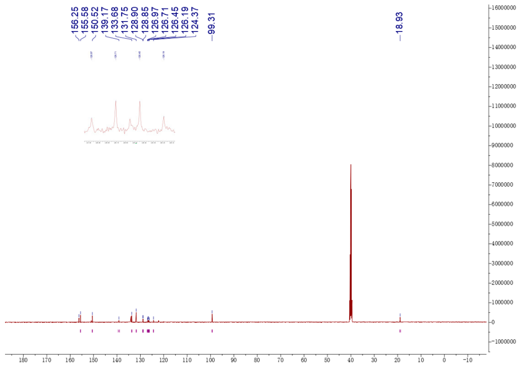

^1H NMR of compound **I-12** ^{13}C NMR of compound **I-12**

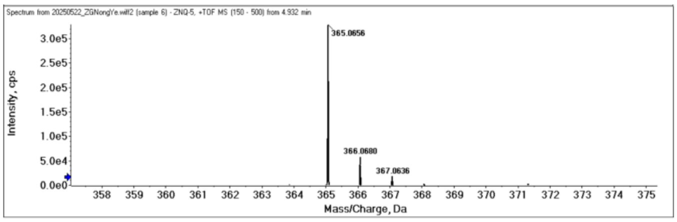

HRSM of compound **I-12**

Data for **I-13. 4-methoxy-N-(5-methyl-7-oxo-1,7-dihydro-[1,2,4]triazolo[1,5-a]pyrimidin-2-yl)benzenesulfonamide.** White solid, m.p.222–223 °C, yield 85%. ^1H NMR (500 MHz, DMSO-d_6) δ 13.15 (s, 1H), 11.60 (s, 1H), 7.90 (d, J = 8.6 Hz, 2H), 7.11 (d, J = 8.7 Hz, 2H), 5.77 (s, 1H), 3.82 (s, 3H), 2.25 (s, 3H). ^{13}C NMR (126 MHz, DMSO-d_6) δ 163.11, 156.65, 155.58, 150.78, 150.52, 132.19, 129.80, 114.68, 99.24, 56.16, 18.93. HRMS(ESI) calcd. for $C_{13}H_{14}N_5O_4S$: $[M+H]^+$ 336.0761, found 336.0761.

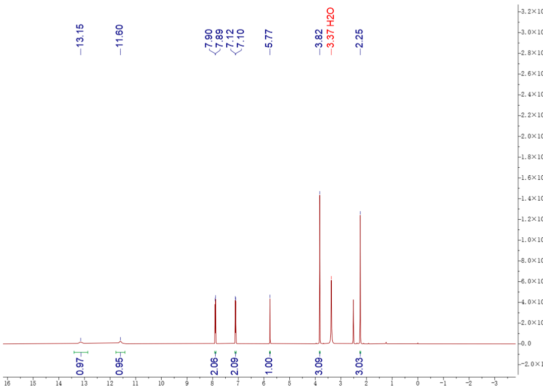

^{1}H NMR of compound **I-13**

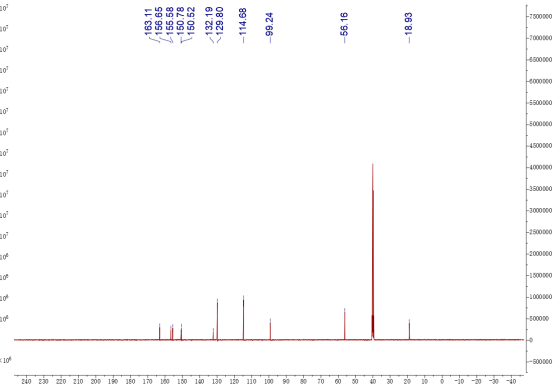

^{13}C NMR of compound **I-13**

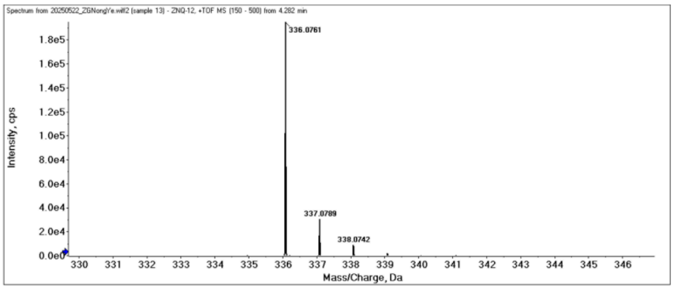

HRMS of compound **I-13**

Data for **I-14. N-(5-methyl-7-oxo-1,7-dihydro-[1,2,4]triazolo[1,5-a]pyrimidin-2-yl)-2-(trifluoromethoxy)benzenesulfonamide**. White solid, m.p.234–235 °C, yield 74%. ^{1}H NMR (500 MHz, DMSO-d$_6$) δ 13.16 (s, 1H), 11.94 (s, 1H), 8.10 – 8.07 (m, 2H), 8.04 – 8.01 (m, 1H), 7.62 (d, J = 8.3 Hz, 2H), 5.78 (s, 1H), 2.24 (s, 3H). ^{13}C NMR (126 MHz, DMSO-d$_6$) δ 156.25, 155.57, 150.98, 150.52, 139.16, 133.99, 133.68, 131.75, 128.90, 128.85, 126.71, 126.45, 124.37, 122.19, 120.02, 99.31, 18.93. HRMS(ESI) calcd. for $C_{13}H_{11}F_3N_5O_4S$: $[M+H]^+$ 390.0478, found 390.0475

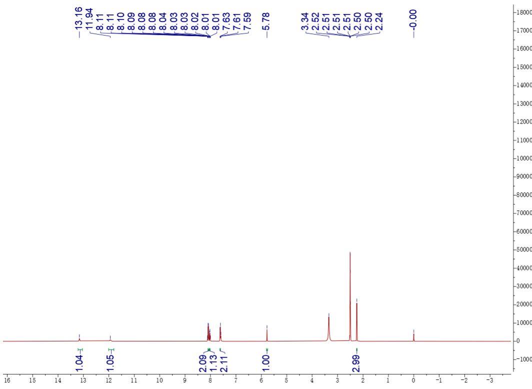

^{1}H NMR of compound **I-14**

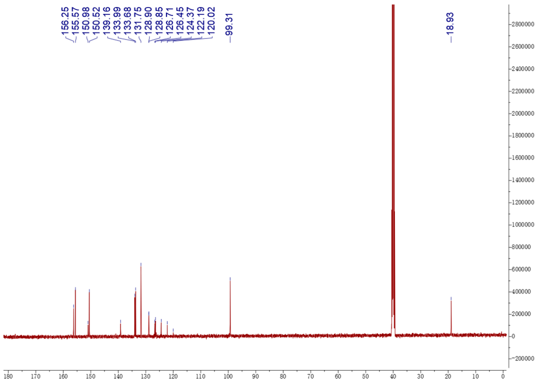

^{13}C NMR of compound **I-14**

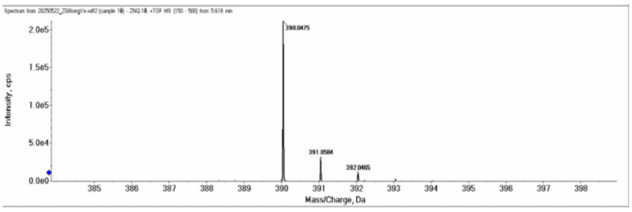

HRMS of compound **I-14**

Data for **I-15. N-(5-methyl-7-oxo-1,7-dihydro-[1,2,4]triazolo[1,5-a]pyrimidin-2-yl)-4-(trifluoromethoxy)benzenesulfonamide**. White solid, m.p.240–241 °C, yield 70%. ^{1}H NMR (500 MHz, DMSO-d_6) δ 13.15 (s, 1H), 12.10 (s, 1H), 8.17 (d, J = 8.1 Hz, 2H), 8.02 (d, J = 8.2 Hz, 2H), 5.78 (s, 1H), 2.24 (s, 3H). ^{13}C NMR (126 MHz, DMSO-d_6) δ 156.18, 155.55, 150.57, 144.56, 133.28, 133.01, 128.49, 126.92, 126.89, 124.97, 122.80, 120.62, 99.38, 18.98. HRMS(ESI) calcd. for $C_{13}H_{11}F_3N_5O_4S$: $[M+H]^+$ 390.0478, found 390.0475.

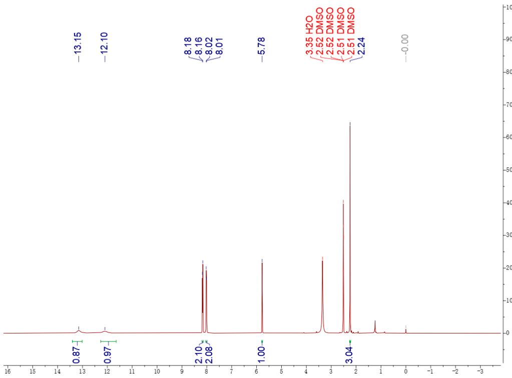

^{1}H NMR of compound **I-15**

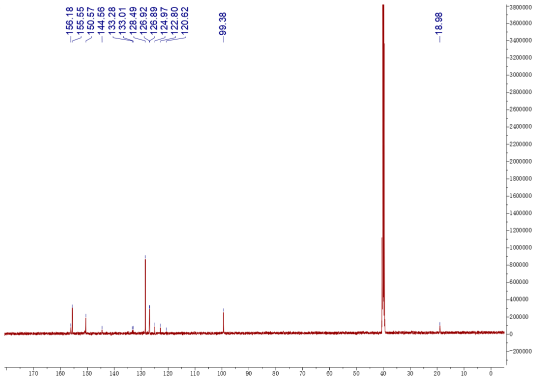

^{13}C NMR of compound **I-15**

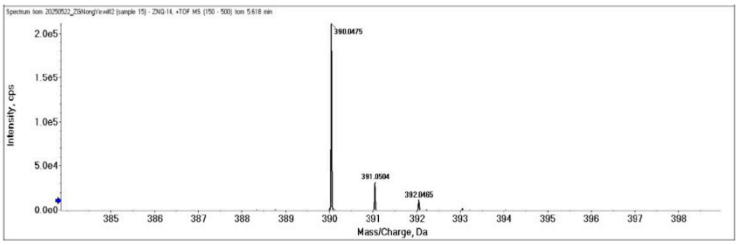

HRMS of compound **I-15**

Data for **I-16. N-(5-methyl-7-oxo-1,7-dihydro-[1,2,4]triazolo[1,5-a]pyrimidin-2-yl)-3-nitrobenzenesulfonamide.** Yellow solid, m.p. 239–240°C, yield 60%. ^{1}H NMR (500 MHz, DMSO-d_6) δ 13.16 (s, 1H), 12.09 (s, 1H), 8.36 (t, J = 1.8 Hz, 1H), 8.25 (dt, J = 8.1, 1.5 Hz, 1H), 8.16 (dt, J = 7.8, 1.4 Hz, 1H), 7.84 (t, J = 7.9 Hz, 1H), 5.78 (s, 1H), 2.24 (s, 3H). ^{13}C NMR (126

MHz, DMSO-d_6) δ 156.07, 155.53, 150.59, 141.82, 137.26, 131.96, 131.25, 131.19, 117.94, 112.78, 99.38, 18.97. HRMS(ESI) calcd. for $C_{12}H_{10}N_6O_5S$: $[M]^+$ 350.0433, found 350.0468.

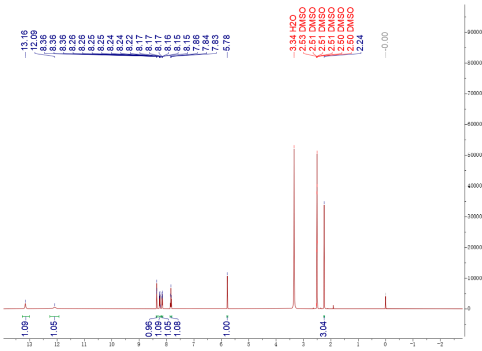

^{1}H NMR of compound **I-16**

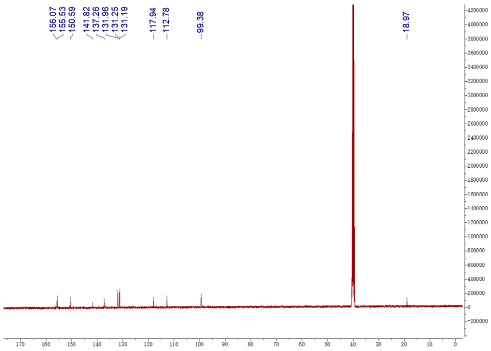

^{13}C NMR of compound **I-16**

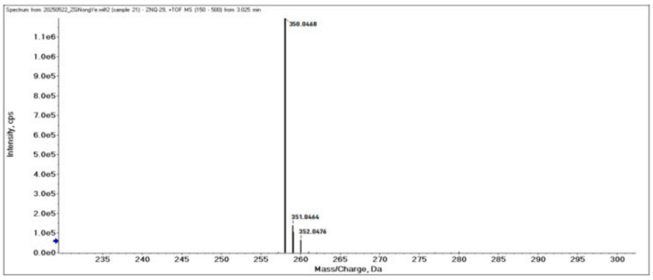

HRMS of compound **I-16**

Data for **I-17. 2,6-difluoro-N-(5-methyl-7-oxo-1,7-dihydro-[1,2,4]triazolo[1,5-a]pyrimidin-2-yl)benzenesulfonamide**. White solid, m.p 210–211. °C, yield 80%. ^{1}H NMR (500 MHz, DMSO-d_6) δ 13.20 (s, 1H), 12.51 (s, 1H), 7.74 (ddd, J = 8.6, 6.0, 2.5 Hz, 1H), 7.31 (t, J = 9.3 Hz, 2H), 5.78 (s, 1H), 2.24 (s, 3H). ^{13}C NMR (126 MHz, DMSO-d_6) δ 160.31, 160.28, 158.26, 158.23, 156.21, 155.56, 151.00, 150.55, 136.29, 118.47, 114.01, 113.98, 113.83, 113.80, 99.34, 18.94. HRMS(ESI) calcd. for $C_{12}H_{10}F_2N_5O_3S$: $[M+H]^+$ 342.0467, found 342.0463.

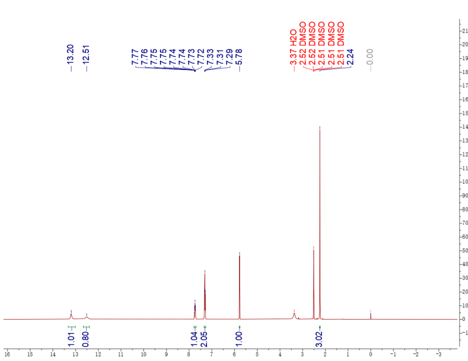

^{1}H NMR of compound **I-17**

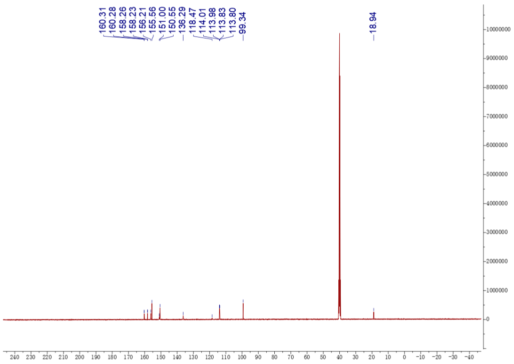

^{13}C NMR of compound **I-17**

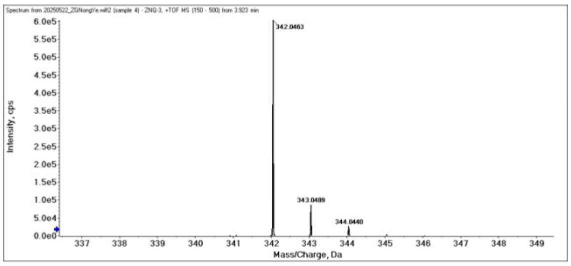

HRMS of compound **I-17**

Data for **I-18. 2,6-dichloro-N-(5-methyl-7-oxo-1,7-dihydro-[1,2,4]triazolo[1,5-a]pyrimidin-2-yl)benzenesulfonamide**. White solid, m.p 250–251. °C, yield 65%. ^{1}H NMR (500 MHz, DMSO-d_6) δ 13.09 (s, 1H), 12.44 (s, 1H), 7.65 (d, J = 8.0 Hz, 2H), 7.57 (dd, J = 8.9, 7.1 Hz, 1H), 5.77 (s, 1H), 2.23 (s, 3H). ^{13}C NMR (126 MHz, DMSO-d_6) δ 156.11, 155.52, 150.95, 150.49, 135.68, 134.67, 134.27, 132.21, 99.34, 18.93. HRMS(ESI) calcd. for $C_{12}H_{10}Cl_2N_5O_3S$: $[M+H]^+$ 373.9873, found 373.9871.

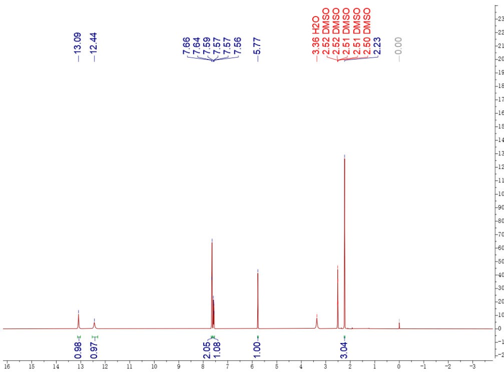

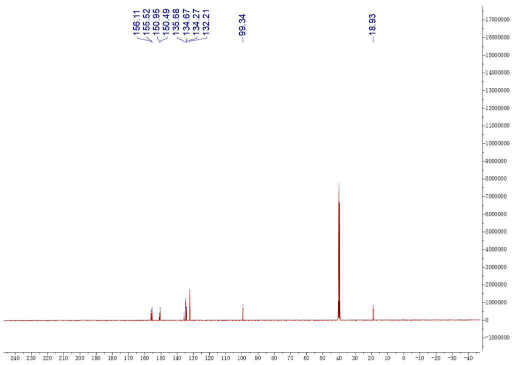

^{1}H NMR of compound **I-18** ^{13}C NMR of compound **I-18**

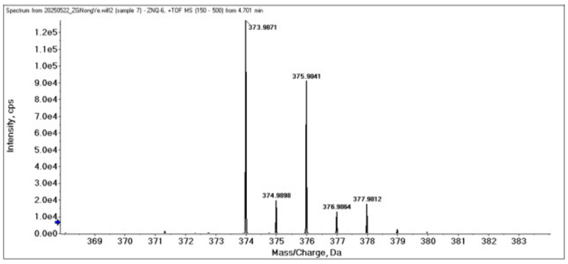

HRMS of compound **I-18**

Data for **I-19. 3,5-dichloro-N-(5-methyl-7-oxo-1,7-dihydro-[1,2,4]triazolo[1,5-a]pyrimidin-2-yl)benzenesulfonamide**. White solid, m.p. 238–239°C, yield 55%. ^{1}H NMR (500 MHz, DMSO-

d$_6$) δ 13.16 (s, 1H), 7.99 (s, 1H), 7.93 (s, 1H), 7.58 (dd, J = 8.2, 4.9 Hz, 1H), 5.87 (s, 1H), 5.78 (s, 1H), 2.25 (s, 3H). ^{13}C NMR (126 MHz, DMSO-d$_6$) δ 155.55, 150.58, 135.33, 133.24, 131.68, 131.61, 126.09, 122.27, 99.46, 19.01. HRMS(ESI) calcd. for $C_{12}H_{10}Cl_2N_5O_3S$: [M+H]$^+$ 373.9876, found 373.9875.

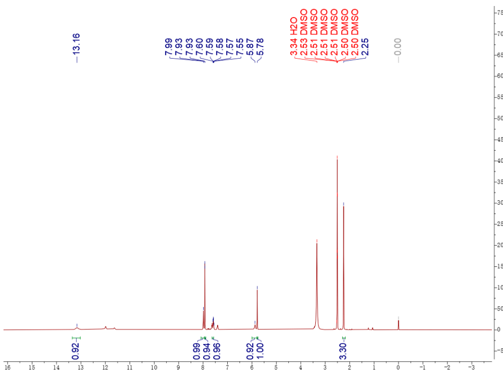

^{1}H NMR of compound **I-19**

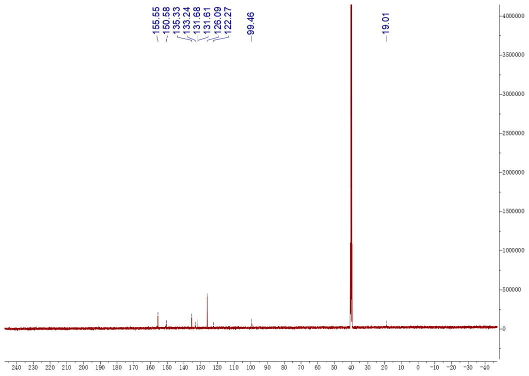

^{13}C NMR of compound **I-19**

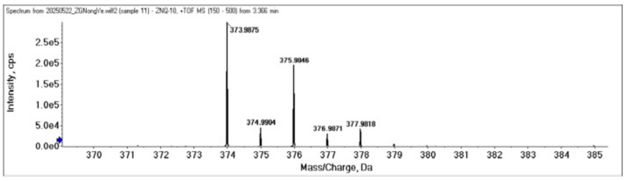

HRMS of compound **I-19**

Data for **I-20. 2-methyl-N-(5-methyl-7-oxo-1,7-dihydro-[1,2,4]triazolo[1,5-a]pyrimidin-2-yl)-5-nitrobenzenesulfonamide**. Yellow solid, m.p.249–250 °C, yield 45%. ^{1}H NMR (500 MHz, DMSO-d$_6$) δ 11.09 (s, 1H), 8.72-8.71 (d, J = 2.6 Hz, 1H), 8.25 – 8.23 (m, 1H), 7.60-7.58 (d, J = 8.4 Hz, 1H), 5.68 (s, 1H), 2.73 (s, 3H), 2.18 (s, 3H). ^{13}C NMR (126 MHz, DMSO-d$_6$) δ 172.49, 155.74, 145.52, 145.16, 133.73, 126.01, 124.20, 99.76, 21.54, 20.56. HRMS(ESI) calcd. for $C_{13}H_{13}N_6O_5S$: [M+H]$^+$ 365.0663, found 365.0656

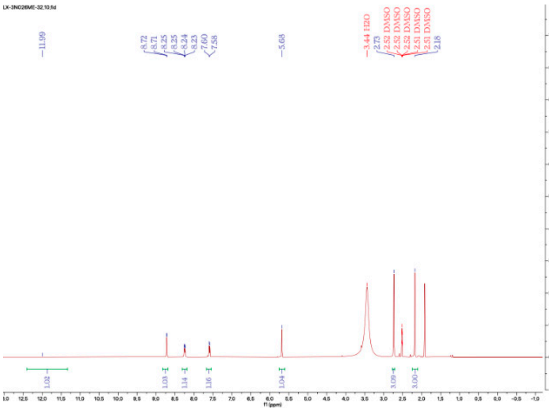

^{1}H NMR of compound **I-20**

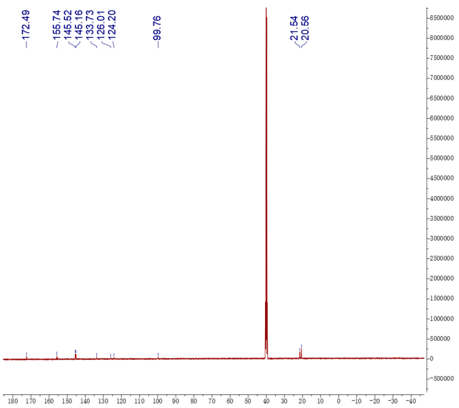

^{13}C NMR of compound **I-20**

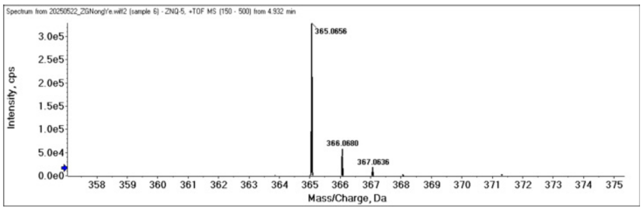

HRMS of compound **I-20**

Data for **I-21. 2-chloro-N-(5-methyl-7-oxo-1,7-dihydro-[1,2,4]triazolo[1,5-a]pyrimidin-2-yl)-5-nitrobenzenesulfonamide.** Yellow solid, m.p.259–260 °C, yield 40%. ^{1}H NMR (500 MHz, DMSO-d_6) δ13.16 (s,1H), 8.55 (d, J = 2.8 Hz, 1H), 8.35 (dd, J = 8.8, 2.8 Hz, 1H), 7.87 (d, J = 8.8 Hz, 1H), 5.64 (s, 1H), 4.13 (s, 1H), 2.23 (s, 3H). ^{13}C NMR (126 MHz, DMSO-d_6) δ 165.35, 163.77, 155.57, 150.60, 146.59, 138.98, 132.87, 132.85, 127.43, 126.11, 98.89, 18.86. HRMS(ESI) calcd. for $C_{12}H_9ClN_6O_5S$: $[M+H]^+$ 384.0116, found 384.0064.

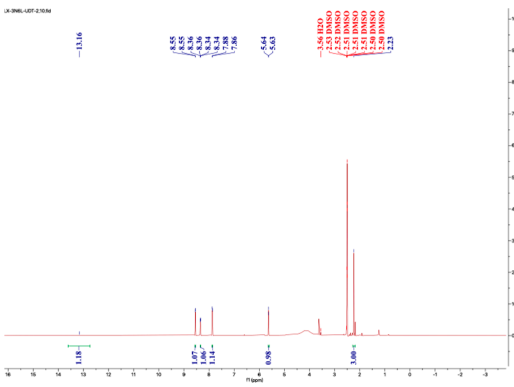

^{1}H NMR of compound **I-21**

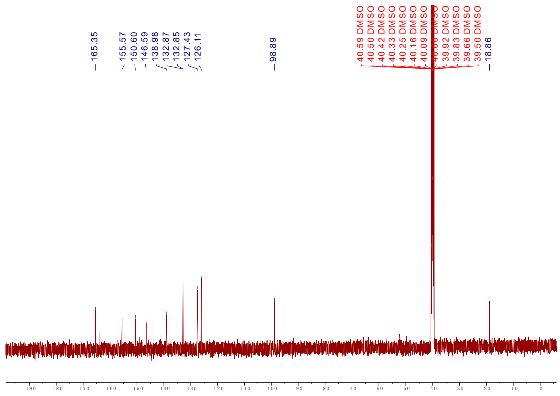

^{13}C NMR of compound **I-21**

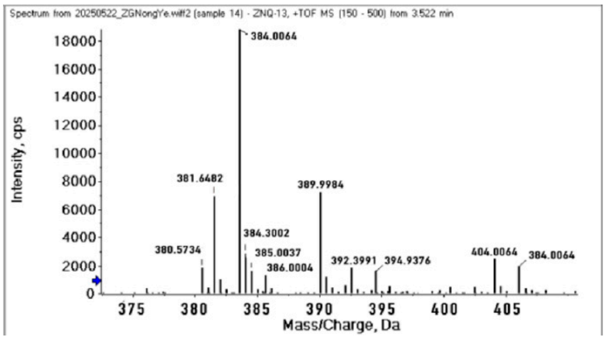

HRMS of compound **I-21**

Data for **I-22. 5-chloro-N-(5-methyl-7-oxo-1,7-dihydro-[1,2,4]triazolo[1,5-a]pyrimidin-2-yl)thiophene-2-sulfonamide**. Dark-white solid, m.p.221–222 °C, yield 65%. ^{1}H NMR (500 MHz, DMSO-d_6) δ 13.15 (s, 1H), 11.89 (s, 1H), 7.95 (d, J = 6.8 Hz, 1H), 7.68 (d, J = 6.7 Hz, 1H), 5.78 – 5.75 (m, 1H), 2.23 (s, 3H). ^{13}C NMR (126 MHz, DMSO-d_6) δ 156.31, 155.55, 150.87, 150.56, 139.44, 138.49, 129.78, 129.48, 99.32, 18.94. HRMS(ESI) calcd. for $C_{10}H_8ClN_5O_3S_2$: $[M+H]^+$ 345.9830, found 345.9826.

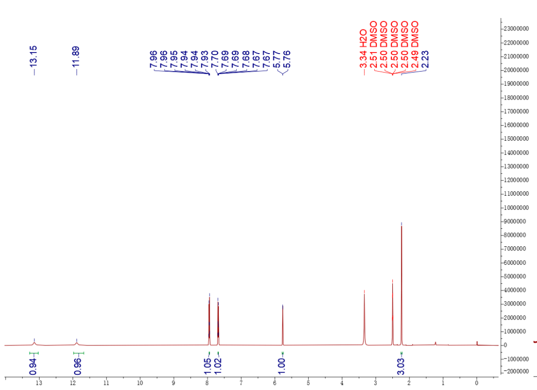

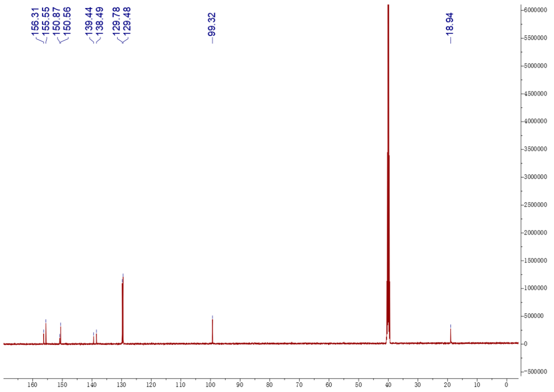

^{1}H NMR of compound **I-22** ^{13}C NMR of compound **I-22**

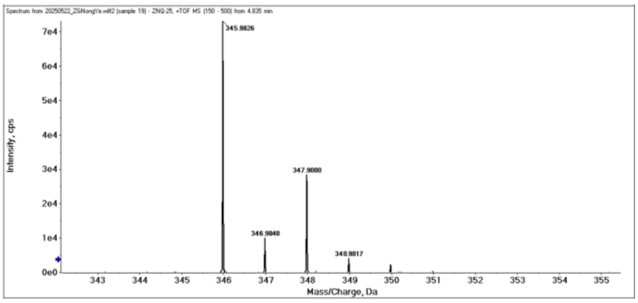

HRMS of compound **I-22**

Data for **I-23. N-(5-(methoxymethyl)-7-oxo-1,7-dihydro-[1,2,4]triazolo[1,5-a]pyrimidin-2-yl)benzenesulfonamide.** Light-brown solid, m.p.198.5–199.5 °C, yield 80%. ^{1}H NMR (500 MHz, DMSO-d_6) δ 7.78 (t, J = 8.3 Hz, 1H), 7.55 (d, J = 3.1 Hz, 1H), 7.39 (d, J = 8.3 Hz, 2H), 7.35 (dd, J

= 9.1, 3.1 Hz, 1H), 6.83 (d, J = 9.0 Hz, 1H), 6.00 (s, 2H), 3.83 (s, 3H). ^{13}C NMR (126 MHz, DMSO-d_6) δ 156.15, 155.58, 150.48, 136.13, 131.83, 129.84, 126.45, 122.24, 99.46, 79.37, 21.53, 19.40. HRMS(ESI) calcd. for $C_{13}H_{13}N_5O_4S$: $[M+H]^+$ 336.0761, found 336.0756.

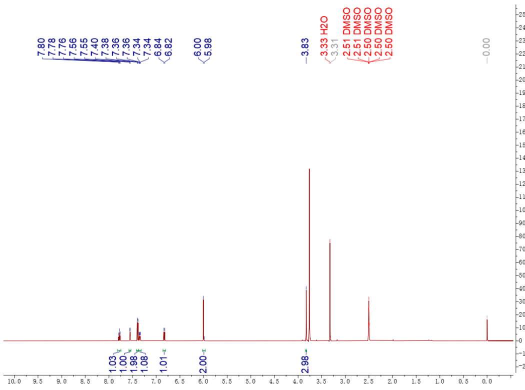

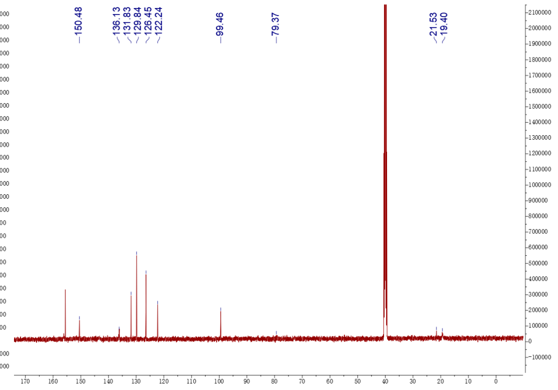

^{1}H NMR of compound **I-23**

^{13}C NMR of compound **I-23**

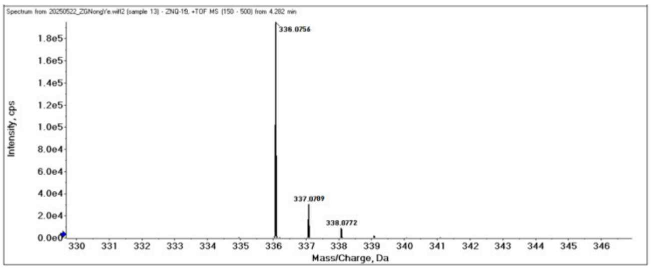

HRMS of compound **I-23**

Data for **I-24. 2-fluoro-N-(5-(methoxymethyl)-7-oxo-1,7-dihydro-[1,2,4]triazolo[1,5-a]pyrimidin-2-yl)benzenesulfonamide**. Dark-white solid, m.p.200–201 °C, yield 85%. ^{1}H NMR (500 MHz, DMSO-d_6) δ 13.92 (s, 1H), 13.13 (s, 1H), 8.31 (d, J = 7.7 Hz, 1H), 7.88 (t, J = 9.3 Hz, 3H), 6.48 (s, 1H), 4.89 (s, 2H), 3.90 (s, 3H). ^{13}C NMR (126 MHz, DMSO-d_6) δ 158.12, 157.45, 152.85, 152.39, 141.04, 135.86, 135.55, 133.62, 130.77, 130.72, 128.84, 128.58, 128.32, 128.06, 126.24, 124.06, 101.18, 60.00, 20.80. HRMS(ESI) calcd. for $C_{13}H_{12}FN_5O_4S$: $[M+H]^+$ 354.0667, found 354.0633

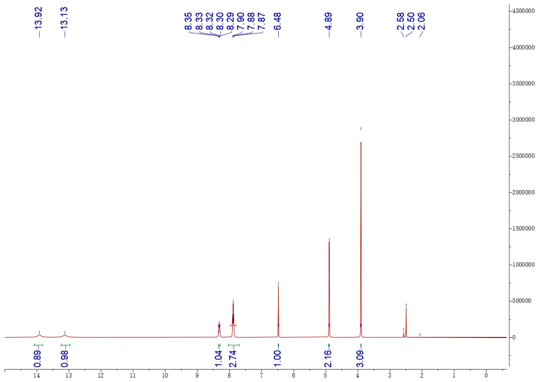

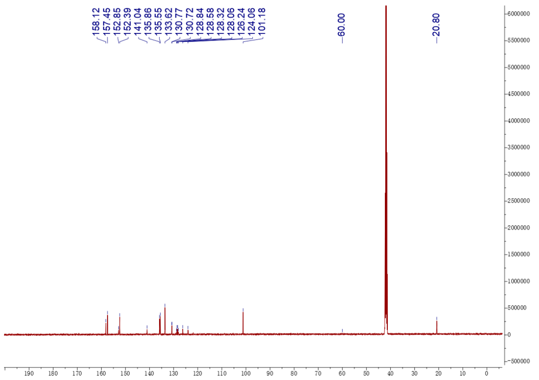

^{1}H NMR of compound **I-24** ^{13}C NMR of compound **I-24**

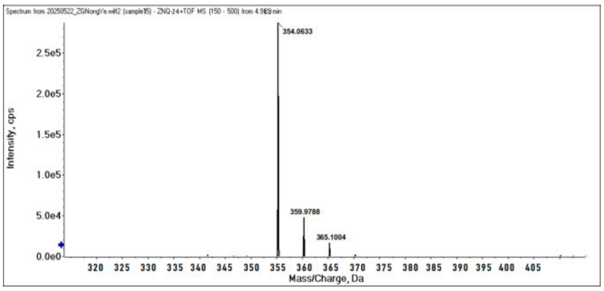

HRMS of compound **I-24**

Data for **I-25. 3-bromo-N-(5-(methoxymethyl)-7-oxo-1,7-dihydro-[1,2,4]triazolo[1,5-a]pyrimidin-2-yl)benzenesulfonamide**. Dark-white solid, m.p. 205–206℃, yield 80%. ^{1}H NMR (500 MHz, DMSO-d_6) δ 12.94 (s, 1H), 12.05 (s, 1H), 8.08 (d, J = 1.9 Hz, 1H), 7.94 (d, J = 7.9 Hz, 1H), 7.86 (d, J = 8.0 Hz, 1H), 7.56 (t, J = 8.0 Hz, 1H), 5.76 (s, 1H), 4.45 (s, 2H), 2.23 (s, 3H). ^{13}C NMR (126 MHz, DMSO-d_6) δ 196.02, 156.15, 155.58, 150.48, 136.13, 131.83, 129.84, 126.45, 122.24, 99.46, 21.53. HRMS(ESI) calcd. for $C_{12}H_{10}BrN_5O_3S$: $[M+2]^+$ 384.9833, found 384.9787.

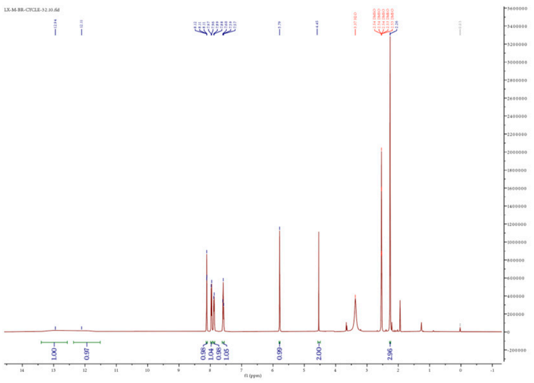

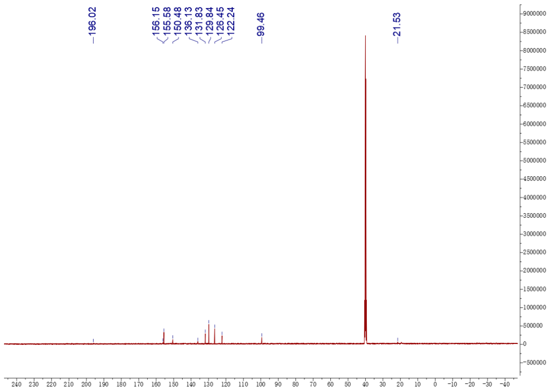

^{1}H NMR of compound **I-25** ^{13}C NMR of compound **I-25**

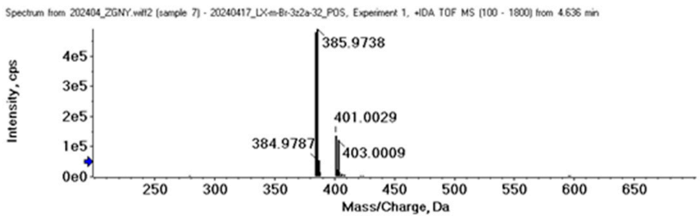

HRMS of compound **I-25**

Data for **I-26. N-(5-(methoxymethyl)-7-oxo-1,7-dihydro-[1,2,4]triazolo[1,5-a]pyrimidin-2-yl)-2-(trifluoromethyl)benzenesulfonamide.** Dark-white solid, m.p.200–201℃, yield 80%. ^{1}H NMR (500 MHz, DMSO-d_6) δ 14.41(s), δ 13.62 (s), δ 8.80 (d, J = 7.7 Hz, 1H), 8.37 (t, J = 9.3 Hz, 3H), 6.97 (s, 1H), 5.38 (s, 2H), 4.39 (s, 3H). ^{13}C NMR (126 MHz, DMSO-d_6) δ 156.25, 155.58, 150.98, 150.52, 139.17, 133.99, 133.68, 131.75, 128.90, 128.85, 126.97, 126.71, 126.45, 126.19, 124.37, 122.19, 99.31, 58.13, 18.93. HRMS(ESI) calcd. for $C_{14}H_{13}F_3N_5O_4S$: $[M+H]^+$ 404.0635, found 404.0638.

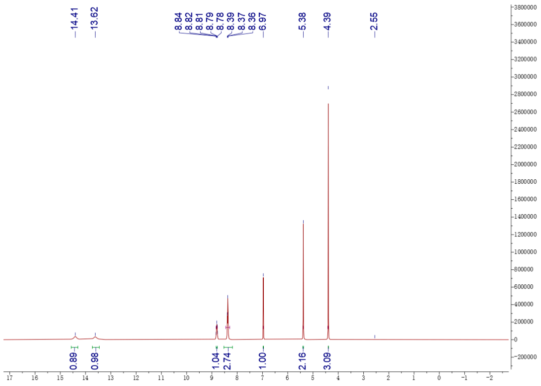

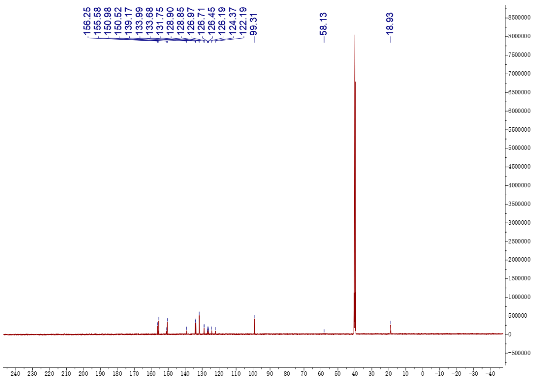

^{1}H NMR of compound **I-26** ^{13}C NMR of compound **I-26**

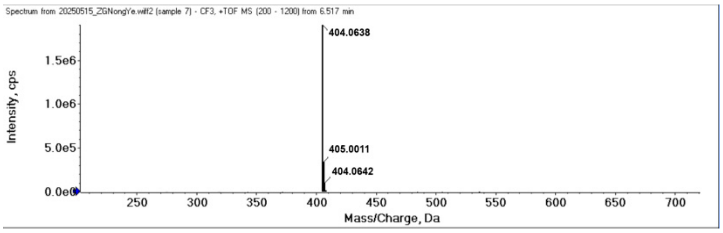

HRMS of compound **I-26**

Data for **I-27. 2,6-difluoro-N-(5-(methoxymethyl)-7-oxo-1,7-dihydro-[1,2,4]triazolo[1,5-a]pyrimidin-2-yl)benzenesulfonamide.** Dark-white solid, m.p. 210.5–211.5℃, yield 65%. 1H NMR (500 MHz, DMSO-d_6) δ 13.33(s), δ 12.54(s), δ 7.73 (q, J = 7.2 Hz, 1H), 7.30 (t, J = 9.3 Hz,

2H), 5.89 (s, 1H), 4.31 (s, 2H), 3.32 (s, 3H). 13C NMR (126 MHz, DMSO-d_6) δ 172.48, 160.33, 160.31, 158.29, 158.26, 156.35, 155.63, 150.67, 136.28, 113.99, 113.82, 113.80, 98.27, 58.70, 21.54. HRMS(ESI) calcd. for $C_{13}H_{11}F_2N_5O_4S$: $[M]^+$ 371.0500, found 371.0528.

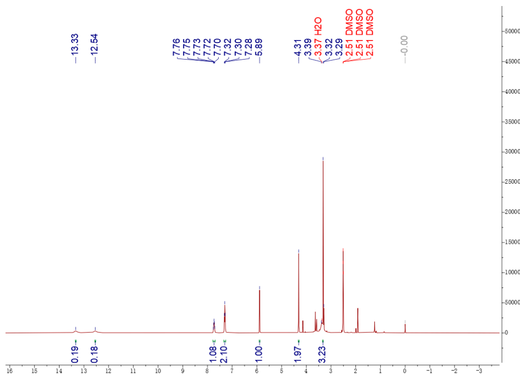

^{1}H NMR of compound **I-27**

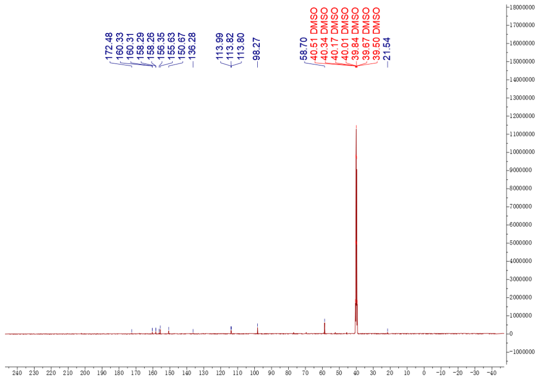

^{13}C NMR of compound **I-27**

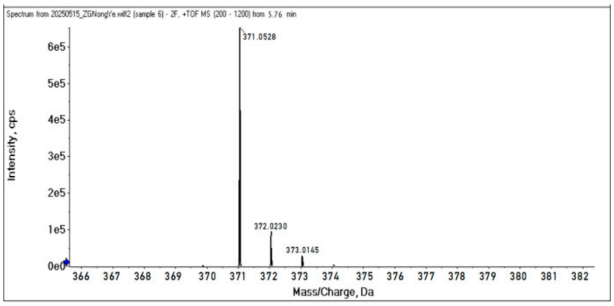

HRMS of compound **I-27**

Data for **I-28. 2,6-dichloro-N-(5-(methoxymethyl)-7-oxo-1,7-dihydro-[1,2,4]triazolo[1,5-a]pyrimidin-2-yl)benzenesulfonamide**. Dark-white solid, m.p. 219–220 °C, yield 75%. ^{1}H NMR (500 MHz, DMSO-d_6) δ 13.33 (s, 1H), 12.54 (s, 1H), 7.73 (q, J = 7.2 Hz, 1H), 7.30 (t, J = 9.3 Hz, 2H), 5.89 (s, 1H), 4.31 (s, 2H), 3.32 (s, 3H). ^{13}C NMR (126 MHz, DMSO-d_6) δ 172.48, 167.97, 160.30, 158.25, 155.63, 150.67, 136.28, 113.80, 98.27, 59.05, 21.54. HRMS(ESI) calcd. for $C_{13}H_{11}Cl_2N_5O_4S$: $[M]^+$ 402.9909, found 402.9912.

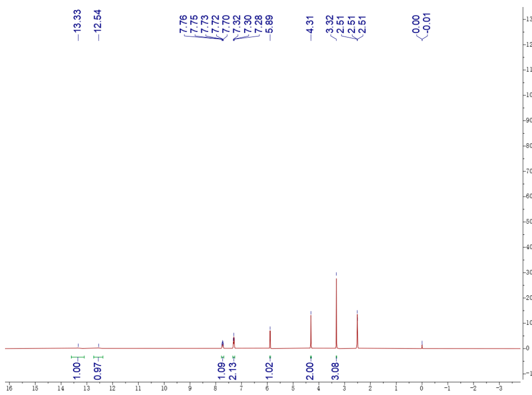

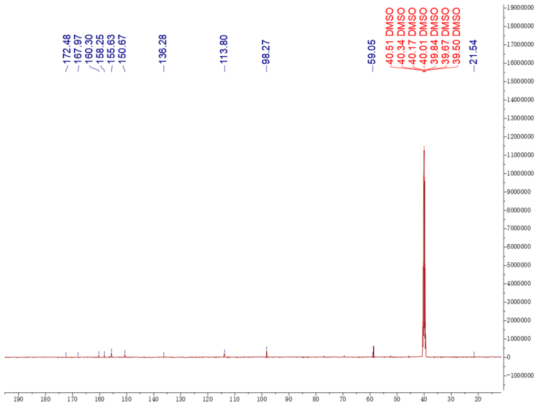

^{1}H NMR of compound **I-28**

^{13}C NMR of compound **I-28**

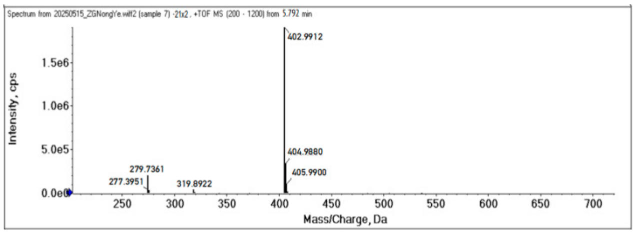

HRMS of compound **I-28**

Data for **I-29. N-(7-(tert-butoxy)-5-methyl-[1,2,4]triazolo[1,5-a]pyrimidin-2-yl)-2-methyl-5-nitrobenzenesulfonamide**. Yellow solid, m.p.267–268 °C, yield 30%. ^{1}H NMR (500 MHz, DMSO-d$_6$) δ 8.52 (d, J = 2.7 Hz, 1H), 8.09 (dd, J = 8.4, 2.6 Hz, 1H), 7.46 (d, J = 8.3 Hz, 1H), 6.96 (brs, 1H), 5.65 (s, 1H), 2.66 (s, 3H), 2.24 (s, 3H), 1.11 (s, 9H).^{13}C NMR (126 MHz, DMSO-d$_6$) δ 163.60, 155.55, 150.55, 149.32, 148.17, 145.33, 144.74, 132.64, 123.73, 121.69, 98.93, 67.38, 31.76, 20.65, 18.85.HRMS(ESI) calcd. for $C_{17}H_{21}N_6O_5S$: $[M+H]^+$ 421.1289, found 421.1288.

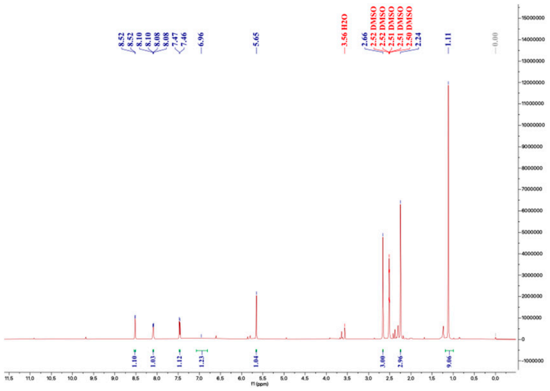

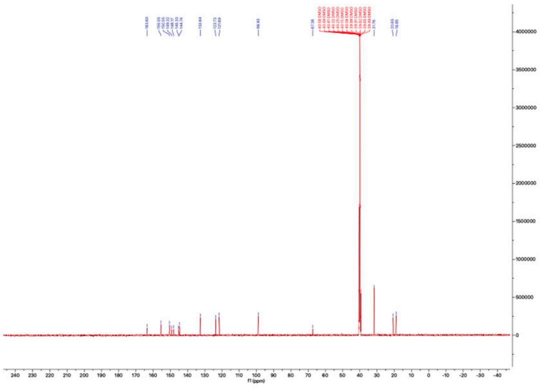

^{1}H NMR of compound **I-29**

^{13}C NMR of compound **I-29**

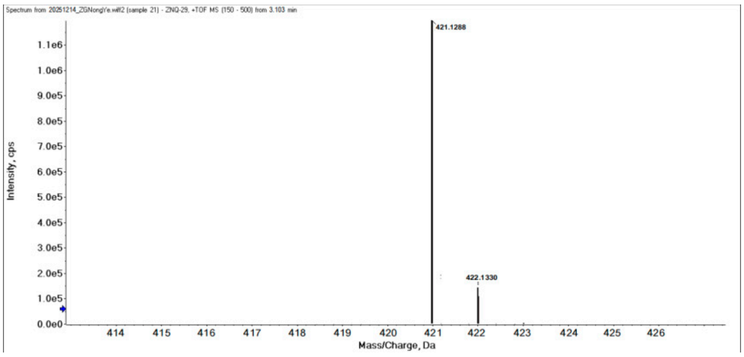

HRMS of compound **I-29**

REFERENCES:

(1) Ram, V. J.; Pratibha, S.; Singh, S. K.; Kandpal, M.; Tekwani, B. L. Functionalized Azoles and Triazolo[1,5-a]Pyrimidines as Latent Leishmanicides. *Bioorg. Med. Chem. Lett.* **1997**, *7* (8), 1087–1090. https://doi.org/https://doi.org/10.1016/S0960-894X(97)00166-2.

2. Single-Crystal X-ray Diffraction Data for Compound I-09

Detailed Experimental Procedure

Single crystals of compound **I-09** were obtained by slow evaporation of a mixed solvent of dichloromethane and methanol at room temperature. Single-crystal X-ray diffraction (SC-XRD) data collection was performed on a Bruker D8 VENTURE diffractometer (Bruker, Germany) with graphite-monochromated Mo Kα radiation (λ = 0.71073 Å) at 100 K.

Data reduction and absorption correction were performed using the SAINT and SADABS programs, respectively. The crystal structure was solved by the intrinsic phasing method using SHELXT, and refined by full-matrix least-squares on F^2 using SHELXL-2018 within the Olex2 1.5 software package. All non-hydrogen atoms were refined anisotropically, and hydrogen atoms were placed in calculated positions and refined using a riding model.

The crystallographic data for the structure of compound **I-09** have been deposited in the Cambridge Crystallographic Data Centre (CCDC) with the deposition number **2499604**. These data can be obtained free of charge from the CCDC via www.ccdc.cam.ac.uk/data_request/cif.

Table S1.Crystal Data and Structure Refinement Details for **I-09**

Parameter	Value
Empirical formula	$C_{12}H_{10}BrN_5O_3S$
Formula weight	384.22
Temperature / K	100
Crystal system	Triclinic
Space group	P -1

a / Å	8.5989(3)
b / Å	12.1430(4)
c / Å	13.7997(5)
α / °	97.590(3)
β / °	103.103(3)
γ / °	94.634(3)
Volume / $Å^3$	1381.92(9)
Z	4
ρcalc / g·cm^{-3}	1.847
μ / mm^{-1}	3.147
F(000)	768
θ range for data collection / °	2.11 to 26.37
Index ranges	$-10 \leq h \leq 10$, $-15 \leq k \leq 15$, $-17 \leq l \leq 17$
Reflections collected	5659
Independent reflections	5632 (Rint = 0.0409)
Data / restraints / parameters	5632 / 1 / 399
Goodness-of-fit on F^2	1.042
Final R1 indexes [I > 2σ(I)]	0.0409 (4385)
Final wR2 indexes [all data]	0.0984
Largest diff. peak / hole / e·$Å^{-3}$	1.57 / -0.85

Table S2. Selected Bond Lengths (Å) and Bond Angles (°) for **I-09**

Bond	Length / Å	Bond	Angle / °
Br1-C13	1.902(4)	N1-C7-N2	111.2(3)
S1-N1	1.632(3)	S1-N1-C7	122.5(2)
S1-C1	1.758(4)	N2-C8-N3	103.5(3)
N3-C8-N4	126.4(3)	C9-O2-C7	115.8(3)

3. Calculation of Octanol-Water Partition Coefficient (logP)

The octanol-water partition coefficient (logP) of the key compounds and commercial herbicide controls was calculated using ACD/Labs software (free trial version, Advanced Chemistry Development Inc., Toronto, Canada) at 25 °C and neutral pH, based on the fragment addition method. The 3D structures of the compounds were pre-optimized by Marvin Sketch, and the most stable conformation was used for calculation. The calculated logP values are listed in Table S1, which are consistent with the discussion in the main text.

Table S3. Calculated logP values of key compounds and commercial controls

**Compound**	**Calculated logP**	**Standard Deviation (SD)**
**I-20**	**0.59**	**0.78**
**I-29**	**3.18**	**0.82**
**penoxsulam**	**2.87**	**1.3**
**cloransulam-methyl**	**3.52**	**0.79**
**diclosulam**	**2.88**	**0.73**

4. Weed control phenotypes

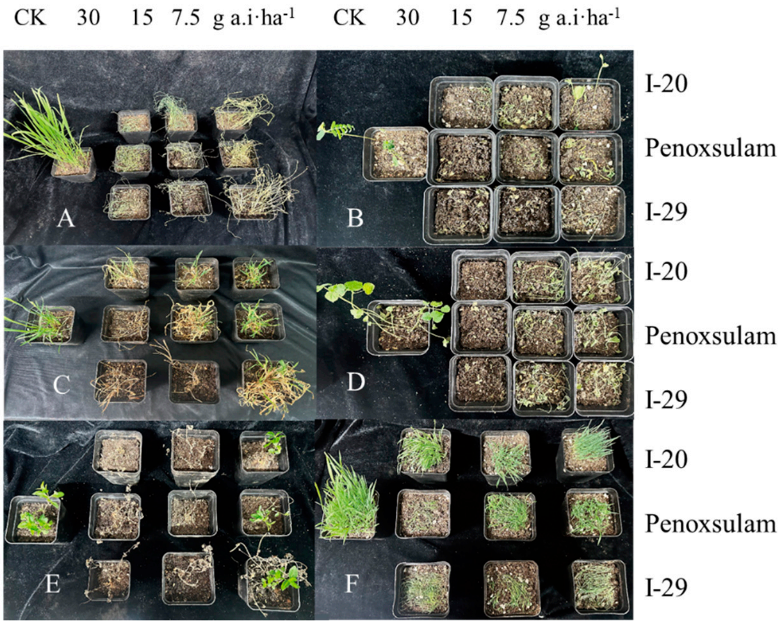

Figure S1. Comparison of herbicidal activity of compounds **I-20**, **I-29** and penoxsulam (A: E. crusgalli; B: S. cannabina; C: E. indica; D: A. theophrasti; E: E. prostrata; F: D. sanguinalis) (taken

fourteen days after treatment).

5. Radar chart of herbicidal activity of compounds at 7.5 g a.i./ha

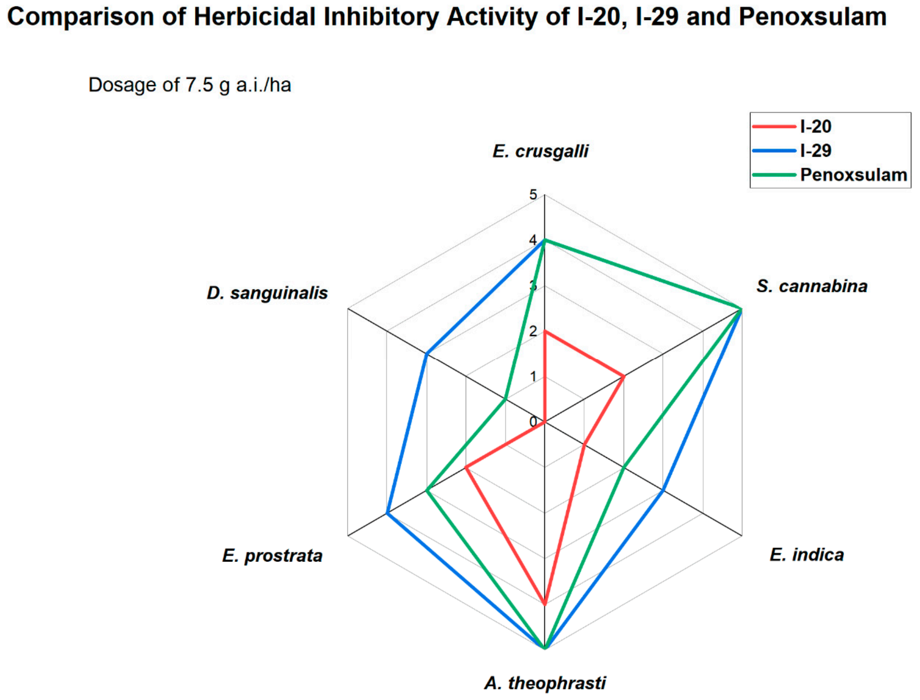

Figure S2. Radar chart of herbicidal activity of **I-20**, **I-29** and penoxsulam at 7.5 g a.i./ha

6. Receptor binding affinity

Table S4. Quantitative Molecular Docking Data of Title Compounds and Commercial Herbicide Controls with Arabidopsis thaliana Acetohydroxyacid Synthase (*At*AHAS)(PDB:5WJ1)

Compound	Pose [a]	Affinity [c] (Kcal/mol)	Estimated K_i [b] [c] (nM)	LE [d] (kcal/mol/atom)	Average binding affinity [f] (Kcal/mol)
**I-20**	1	-9.2	182.35	-0.368	-8.65
	2	-9.2	182.35	-0.368	
	3	-8.7	423.18	-0.348	
	4	-8.7	423.18	-0.348	
	5	-8.6	499.87	-0.344	
	6	-8.6	499.87	-0.344	
	7	-8.6	499.87	-0.344	
	8	-8.5	594.72	-0.340	
	9	-8.3	836.51	-0.332	
	10	-8.1	1182.45	-0.324	
**I-29**	1	-9.6	90.17	-0.331	-8.89
	2	-9.3	153.89	-0.321	
	3	-9.1	215.74	-0.314	
	4	-9	255.68	-0.310	
	5	-8.8	358.92	-0.303	
	6	-8.8	358.92	-0.303	
	7	-8.7	423.18	-0.300	
	8	-8.6	499.87	-0.297	
	9	-8.6	499.87	-0.297	
	10	-8.4	718.34	-0.290	
**Penoxsulam**	1	-9.4	121.56	-0.313	-8.83
	2	-9.2	182.35	-0.307	
	3	-9	255.68	-0.300	
	4	-9	255.68	-0.300	
	5	-8.9	307.25	-0.297	
	6	-8.9	307.25	-0.297	
	7	-8.6	499.87	-0.287	
	8	-8.6	499.87	-0.287	
	9	-8.4	718.34	-0.280	
	10	-8.3	836.51	-0.277	
**Pyroxsulam**	1	-9.3	153.89	-0.332	-8.81
	2	-9.3	153.89	-0.332	
	3	-9.1	215.74	-0.325	
	4	-8.9	307.25	-0.318	
	5	-8.6	499.87	-0.307	
	6	-8.6	499.87	-0.307	

7	-8.6	499.87	-0.307
8	-8.6	499.87	-0.307
9	-8.6	499.87	-0.307
10	-8.5	594.72	-0.304

a. Pose: Refers to the possible binding conformations of the ligand to the AHAS enzyme obtained from molecular docking simulations; the numbers 1–10 represent the serial numbers of different docking-generated binding conformations.

b. Estimated $K_{i:}$ K_i(Inhibition constant) is the equilibrium dissociation constant reflecting the binding affinity between the compound and AHAS enzyme with the unit of nM.

c. The values of Affinity (Kcal/mol) and Estimated Ki (μmol/L) were all derived from molecular docking simulation results, and the numerical precision was retained to 2 decimal places (except for individual integer values).

d. Ligand efficiency (LE) was calculated using the formula: LE = Affinity (Kcal/mol) / Number of non-hydrogen atoms (NHA) of the ligand; the Affinity values were from the corresponding column in this table, and NHA refers to the number of non-hydrogen atoms of the target ligand.

e. All data in the table are the original results of molecular docking simulations with Autodock Vina, presented as the actual simulation outcomes without repeated experiment correction.

f. Average binding affinity (Kcal/mol): Refers to the arithmetic mean of Affinity (Kcal/mol) values corresponding to all binding conformations (Pose) of the same compound, with the same unit (Kcal/mol) as Affinity.
